# Mapping the Substrate-Binding Sites in the Phosphatidylserine Synthase in *Candida albicans*

**DOI:** 10.3389/fcimb.2021.765266

**Published:** 2021-12-22

**Authors:** Yue Zhou, Chelsi D. Cassilly, Todd B. Reynolds

**Affiliations:** Department of Microbiology, University of Tennessee Knoxville, Knoxville, TN, United States

**Keywords:** membrane lipid, phospholipids/biosynthesis, enzyme kinetics, mutagenesis, serine, phosphatidylserine, cytidyldiphosphate-diacylglycerol, CDP-alcohol phosphotransferase

## Abstract

The fungal phosphatidylserine (PS) synthase, a membrane protein encoded by the *CHO1* gene, is a potential drug target for pathogenic fungi, such as *Candida albicans*. However, both substrate-binding sites of *C. albicans* Cho1 have not been characterized. Cho1 has two substrates: cytidyldiphosphate-diacylglycerol (CDP-DAG) and serine. Previous studies identified a conserved CDP-alcohol phosphotransferase (CAPT) binding motif, which is present within Cho1. We tested the CAPT motif for its role in PS synthesis by mutating conserved residues using alanine substitution mutagenesis. PS synthase assays revealed that mutations in all but one conserved amino acid within the CAPT motif resulted in decreased Cho1 function. In contrast, there were no clear motifs in Cho1 for binding serine. Therefore, to identify the serine binding site, PS synthase sequences from three fungi were aligned with sequences of a similar enzyme, phosphatidylinositol (PI) synthase, from the same fungi. This revealed a motif that was unique to PS synthases. Using alanine substitution mutagenesis, we found that some of the residues in this motif are required for Cho1 function. Two alanine substitution mutants, L184A and R189A, exhibited contrasting impacts on PS synthase activity, and were characterized for their Michaelis-Menten kinetics. The L184A mutant displayed enhanced PS synthase activity and showed an increased *V*_max_. In contrast, R189A showed decreased PS synthase activity and increased *K*_m_ for serine, suggesting that residue R189 is involved in serine binding. These results help to characterize PS synthase substrate binding, and should direct rational approaches for finding Cho1 inhibitors that may lead to better antifungals.

## Introduction

*Candida* spp. are the most common causes of fungal infections in humans and are a major cause of fungi-associated mortality worldwide (Brown et al., 2012). These species are versatile pathogens, with *C. albicans* being the most common. While they can infect almost all body sites, they are most prevalently seen in infections of the oral mucosa, vaginal mucosa, and bloodstream/deep organs (*i.e.*, invasive mycoses) (Kullberg et al., 2002). The latter is of greatest concern because they are associated with a high (~40%) mortality rate (Wisplinghoff et al., 2004; Morrell et al., 2005). There are only three classes of antifungals that are commonly used to treat systemic infections: echinocandins (e.g., caspofungin), azoles (e.g., itraconazole), and polyenes (e.g., amphotericin B) (Pfaller et al., 2012). However, these all have limitations, which include drug resistance for the echinocandins and azoles and patient toxicity for the polyene amphotericin B (Holeman Jr and Einstein, 1963; Ghannoum and Rice, 1999; Odds et al., 2003; Whaley et al., 2016). Thus, there is an urgent need to discover new drugs. One classic approach is to identify virulence-related proteins within *C. albicans* that are not conserved in humans and exploit them as drug targets.

The phosphatidylserine (PS) synthase in *C. albicans* represents a potential drug target for three reasons: 1) It is required for virulence (Chen et al., 2010), indicating that inhibitors of this enzyme would render the organism incapable of causing infection; 2) it is absent in humans (Braun et al., 2005), so inhibitors potentially would have no toxic side effects for the host; and 3) it is conserved among many fungi, so a drug could potentially be effective against multiple fungal pathogens. Recently, deletion of PS synthase in *Cryptococcus neoformans* was shown to be lethal, suggesting phosphatidylserine synthesis is also essential for the viability of some fungi (Konarzewska et al., 2019). This observation further strengthens the potential of PS synthase as a broad antifungal drug target.

The PS synthase enzyme (originally denoted as: cytidine 5’-diphospho-1,2-diacyl-sn-glycerol: l-serine O-phosphatidyltransferase, gene name: *CHO1*) was first identified in *Saccharomyces cerevisiae* (Atkinson et al., 1980a; Atkinson et al., 1980b; Kovac et al., 1980). Since then, characterization of *S. cerevisiae* Cho1 included protein solubilization and purification (Carman and Matas, 1981; Bae-Lee and Carman, 1984), determination of Michaelis-Menton kinetics (Carman and Matas, 1981; Carson et al., 1982; Bae-Lee and Carman, 1984), understanding regulation of Cho1 (Carson et al., 1982; Poole et al., 1986; Bailis et al., 1987; Kelley et al., 1988; Kinney and Carman, 1988), and identifying the localization of the enzyme (Kuchler et al., 1986; Kohlwein et al., 1988). The function of the first fungal pathogen Cho1 homolog was described in *C. albicans* and this protein was shown to be required for both systemic and oral *Candida* infection in the mouse model (Chen et al., 2010; Davis et al., 2018). Later, the Michaelis Menten kinetics of the wildtype *C. albicans* Cho1 were biochemically determined, which yielded a millimolar-scale *K* _m_ for serine, similar to that reported for *S. cerevisiae* Cho1 (Carman and Matas, 1981; Cassilly et al., 2017). These findings again highlight the enzyme’s potential as a drug target for *Candida* infections.

The long-term goal of characterizing Cho1 is to discover a small molecule inhibitor of *C. albicans* Cho1 that can be used as a lead compound for drug development. Small molecule screening is a very effective approach to identify inhibitors of enzymes, but another strategy is to use a rational approach for identifying inhibitors (Mandal et al., 2009). Ligand-based drug design for *C. albicans* Cho1 is limited because of the small number of known PS synthase ligands or ligand analogs, as well as the ubiquitous nature of its natural substrates. In addition, neither the three-dimensional structure nor the binding sites for substrates of *C. albicans* Cho1 are available, which hinders structure-based design. Thus, it is critical to the foundation of these approaches that the binding sites for the two substrates, cytidyldiphosphate-diacylglycerol (CDP-DAG) and serine, are described. Here, we biochemically identified potential substrate binding motifs in the enzyme. Identification of these sites in the protein will facilitate a more directed approach to discovering small molecules that might interact with *C. albicans* Cho1.

Previously, a highly conserved motif, D-(X)_2_-D-G-(X)_2_-A-R-(X)_2_-N-(X)_5_-G-(X)_2_-L-D-(X)_3_-D, was identified from the alignment of yeast phosphatidylinositol synthase (PI synthase), phosphatidylserine synthase, and the *E. coli* phosphatidylglycerophosphate synthase (PGP synthase) (Nikawa et al., 1987). This motif has been further identified in yeast cholinephosphotransferase (Cpt1) and ethanolaminephosphotransferase (Ept1) (Hjelmstad and Bell, 1990; Hjelmstad and Bell, 1991). All of these enzymes bind a CDP-linked molecule and a second small alcohol and catalyze the formation of a phosphodiester bond between the two. Since these initial studies, this CDP-alcohol phosphatidyltransferase (CAPT) motif was narrowed to D-G-(X)_2_-A-R-(X)_8_-G-(X)_3_-D-(X)_3_-D, and was identified to be almost invariably conserved in numerous other lipid biosynthetic enzymes, including those of Gram positive and Gram negative bacteria, archaea, fungi, plants, and mammals (Dryden and Dowhan, 1996; Tanaka et al., 1996; Matsumoto, 1997; Delhaize et al., 1999; Matsuo et al., 2007; Grāve et al., 2019; Konarzewska et al., 2019). The CAPT motif in yeast Cpt1 was characterized by alanine substitution mutagenesis, providing information on the importance of specific residues within the conserved motif (Williams and McMaster, 1998).

More recently, two enzymes from the CDP-alcohol phosphatidyltransferase family from *Archaeoglobus fulgidus* were shown to contain this motif on helices TM2 and TM3 of the solved crystal structures (Nogly et al., 2014; Sciara et al., 2014). In these studies, the CAPT motif was also widened to include an extra aspartic acid, generating the current, more general motif: D_1_xxD_2_G_1_xxAR … G_2_xxxD_3_xxxD_4_ (**Table 1**). This was further confirmed in the phosphatidylinositol-phosphate synthase from *Renibacterium salmoninarum*, *Mycobacterium tuberculosis*, and *Mycobacterium kansasii*, where the CAPT motif was again found within TM2 and TM3 of the solved crystal structures (Clarke et al., 2015; Grāve et al., 2019; Dufrisne et al., 2020). To our knowledge, these are the five CDP-alcohol phosphatidyltransferase enzymes with solved crystal structures, and there is currently no eukaryotic counterpart solved.

**Table 1:** The CAPT motif for binding CDP-alcohols is mostly conserved across domains.

Organism	Enzyme	CDP-Binding Motif*	Reference
*C. albicans*	PS Synthase	_125_DFFDGRVARLRNKSSLMGQELDSLAD_150_	This research
*S. cerevisiae*	PS Synthase	_127_DFFDGRVARLRNKSSLMGQELDSLAD_152_	(Kiyono et al., 1987)
*S. cerevisiae*	Cholinephospho-transferase (Cpt1)	_110_DMHDGMHARRTGQQGPLGELFDHCID_135_	(Williams and McMaster, 1998)
*S. pombe*	PS Synthase	_92_DFLDGKVARWRGKSSLMGQELDSLAD_117_	(Matsuo et al., 2007)
*B. subtilis*	PS Synthase	_42_DFFDGMAARKLNAVSDMGRELDSFAD_67_	(Okada et al., 1994)
*S. meliloti*	Phosphatidylcholine (PC) Synthase	_56_DGIDGPIARKVQVKEVLPNWSGDTLDNVID_85_	(Sohlenkamp et al., 2000)
*A. fulgidus*	CDP-alcohol phosphotransferase AF2299	_214_DGCDGEIARLKFMESKYGAWLDGVLD_239_	(Sciara et al., 2014)
*A. fulgidus*	CDP-alcohol phosphotransferase IPCT-DIPPS	_357_DGCDGEIARASLKMSKKGGYVDSILD_382_	(Nogly et al., 2014)
*R. salmoninarum*	PIP synthase	_66_DIIDGLMARLLFREGPWGAFLDSYLD_91_	(Clarke et al., 2015)
*M. tuberculosis*	PIP synthase	_68_DMLDGAMARERGGGTRFGAVLDATCD_93_	(Grāve et al., 2019)
*M. kansasii*	PIP synthase	_68_DMLDGAMARLRSGGTRFGAVLDAACD_93_	(Dufrisne et al., 2020)
*C. albicans* PS Synthase		DFFDGRVARLRNKS—SLMGQELDSLAD	
*S. cerevisiae* PS Synthase		DFLDGRVARLRNRS—SLMGQELDSLAD	
*S. cerevisiae* Cpt1		DMHDGMHARRTGQQ—GPLGELFDHCID	
*S. pombe* PS Synthase		DFLDGKVARWRGKS—SLMGQELDSLAD	
*B. subtilis* PS Synthase		DFFDGMAARKLNAV—SDMGRELDSFAD	
*S. meliloti* PC Synthase		DGIDGPIARKVQVKEVLPNWSGDTLDNVID	
*A. fulgidus* AF2299		DGCDGEIARLKFME—SKYGAWLDGVLD	
*A. fulgidus* IPCT-DIPPS		DGCDGEIARASLKM—SKKGGYVDSILD	
*R. salmoninarum* PIP synthase		DIIDGLMARLLFRE—GPWGAFLDSYLD	
*M. tuberculosis* PIP synthase		DMLDGAMARERGGG—TRFGAVLDATCD	
*M. kansasii* PIP synthase		DMLDGAMARLRSGG—TRFGAVLDAACD	
CAPT motif**		**D** **_1_** **xx D** **_2_** **G**_**1**_ **xx AR ……………………. G** **_2_** **xxx D** **_3_** **xxx D _4_**	

*Gray highlighted residues represent the conserved amino acids. **Bold amino acids represent conserved residues in the CAPT motif.

Based on the above studies, there is sequence homology to guide a search for the CAPT motif in Cho1, but the motif specific for the other substrate, serine, is unknown. In fact, for many of the CDP-DAG binding enzymes, such as PI synthase and PGP synthase, the binding sites for the non-CDP substrates are unclear. Furthermore, some important residues involved in serine binding or recognition from other serine-utilizing enzymes have been identified, but these residues are unlikely to inform our search as these enzymes catalyze very different reactions and the equivalent residues are absent in Cho1 (Ohsawa et al., 2004; Ikushiro et al., 2009). Thus, identification of the serine binding site—or some residues involved in serine binding, even if it is not the full motif—in Cho1 will facilitate a better understanding of this class of enzymes which are crucial for phospholipid biosynthesis in all domains of life.

Previously, we described the apparent *K* _m_ and *V*_max_ for *C. albicans* PS synthase, as well as its role in the phospholipidome of *C. albicans* (Cassilly et al., 2017). Furthermore, we probed the specificity of the active site of this protein for L-serine by competition assays with the closely related amino acids L-threonine and D-serine by an *in vitro* assay (Cassilly et al., 2017). We found that only very high concentrations of these substrates could compete with L-serine, indicating that the enzyme seems to be specific for L-serine, which agrees with previous studies in *S. cerevisiae* and *E. coli* (Kanfer and Kennedy, 1964; Carson et al., 1982). To further reveal insights into the active sites in the present communication, we mapped and characterized residues that affect binding for both substrates in Cho1.

## Materials and Methods

### Strains and Media

In this study, we used the *cho1*∆∆ mutant (YLC337) and *cho1*∆∆*::CHO1* strain created from the SC5314 (wildtype) strain of *C. albicans*, which have been described previously (Chen et al., 2010). The *cho1*∆∆ strain was used to generate the *cho1*∆∆*P_ENO1_-CHO1-HAx3* strain (HA1) and its binding site mutant derivatives (**Table 2**). The media used to culture strains were YPD or minimal medium (0.67% yeast nitrogen base, 2% dextrose) ± 1 mM ethanolamine, where indicated.

**Table 2:** Strains produced in this study.

Organism	Strain	Plasmid	Gene/Mutation	Genotype
*Candida albicans*	HA1	pCDC4	*CHO1-HAx3*	*cho1*∆∆*P_ENO1_-CHO1-HAx3*
*Candida albicans*	CDCS60	pCDC15	*CHO1^D125A^-HAx3*	*cho1*∆∆*P_ENO1_-CHO1^D125A^-HAx3*
*Candida albicans*	CDCS61	pCDC8	*CHO1^D128A^-HAx3*	*cho1*∆∆*P_ENO1_-CHO1^D128A^-HAx3*
*Candida albicans*	CDCS62	pCDC14	*CHO1^G129A^-HAx3*	*cho1*∆∆*P_ENO1_-CHO1^G129A^-HAx3*
*Candida albicans*	CDCS63	pCDC10	*CHO1^R133A^-HAx3*	*cho1*∆∆*P_ENO1_-CHO1^R133A^-HAx3*
*Candida albicans*	CDCS64	pCDC12	*CHO1^G142A^-HAx3*	*cho1*∆∆*P_ENO1_-CHO1^G142A^-HAx3*
*Candida albicans*	CDCS65	pCDC11	*CHO1^D146A^-HAx3*	*cho1*∆∆*P_ENO1_-CHO1^D146A^-HAx3*
*Candida albicans*	CDCS66	pCDC9	*CHO1^D150A^-HAx3*	*cho1*∆∆*P_ENO1_-CHO1^D150A^-HAx3*
*Candida albicans*	YZ 7	pYZ2	*CHO1^V180A^-HAx3*	*cho1*∆∆*P_ENO1_-CHO1^V180A^-HAx3*
*Candida albicans*	YZ 8	pYZ3	*CHO1^L181A^-HAx3*	*cho1*∆∆*P_ENO1_-CHO1^L181A^-HAx3*
*Candida albicans*	YZ 9	pYZ4	*CHO1^C182A^-HAx3*	*cho1*∆∆*P_ENO1_-CHO1^C182A^-HAx3*
*Candida albicans*	YZ 10	pYZ5	*CHO1^G183A^-HAx3*	*cho1*∆∆*P_ENO1_-CHO1^G183A^-HAx3*
*Candida albicans*	YZ 11	pYZ6	*CHO1^L184A^-HAx3*	*cho1*∆∆*P_ENO1_-CHO1^L184A^-HAx3*
*Candida albicans*	CDCS67	pCDC23	*CHO1^R186A^-HAx3*	*cho1*∆∆*P_ENO1_-CHO1^R186A^-HAx3*
*Candida albicans*	CDCS68	pCDC24	*CHO1^L187A^-HAx3*	*cho1*∆∆*P_ENO1_-CHO1^L187A^-HAx3*
*Candida albicans*	CDCS69	pCDC22	*CHO1^R189A^-HAx3*	*cho1*∆∆*P_ENO1_-CHO1^R189A^-HAx3*
*Candida albicans*	CDCS70	pCDC25	*CHO1^F190A^-HAx3*	*cho1*∆∆*P_ENO1_-CHO1^F190A^-HAx3*
*Candida albicans*	YZ32	pYZ32	*CHO1^R133E^-HAx3*	*cho1*∆∆*P_ENO1_-CHO1^R133E^-HAx3*
*Candida albicans*	YZ57	pYZ50	*CHO1^G129P^-HAx3*	*cho1*∆∆*P_ENO1_-CHO1^G129P^-HAx3*

### Genetic Cloning and Site-Directed Mutagenesis

The plasmid containing *CHO1-HAx3* is called pCDC31 and was generated as follows: The plasmid pBT1, containing the *ENO1* promoter (*P_ENO1_*) and the *SAT1* marker (Tams et al., 2019), was used as a vector. The *CHO1* gene was amplified from SC5314 genomic DNA using primers CCO160, which sits upstream of the *CHO1* start site and includes a 5’ *NotI* cut site, and CCO55 which sits at the 3’ end of *CHO1* just upstream of the stop codon and includes a 3’ 2x HA tag followed by an *AatII* cut site. The 3’ untranslated region (3’UTR) of *CHO1* was amplified using CCO56, which sits immediately downstream of the *CHO1* stop codon and contains a 5’ *AatII* cut site followed by a 1x HA tag before the downstream sequence, and CCO163, which sits 500 bp downstream of the *CHO1* stop codon and includes a 3’ *SacI* cut site. The 3’UTR was included in the construct to increase the stability of the transcripts (Mignone et al., 2002). Once amplified, both fragments were double digested with their respective enzyme combinations. The plasmid pBT1 was digested with *NotI* and *SacI* and all three fragments were ligated together to create pCDC31. All plasmids are listed in **Table 3**.

**Table 3:** Plasmids used in this study.

Plasmid	Inserts*	Source
pYLC314-TBR	NAT^R^, Amp^R^	(Tams et al., 2019)
pCDC31	NAT^R^, Amp^R^, *CHO1*	This study
pCDC15	NAT^R^, Amp^R^, *CHO1^D125A^*	This study
pCDC8	NAT^R^, Amp^R^, *CHO1^D128A^*	This study
pCDC14	NAT^R^, Amp^R^, *CHO1^G129A^*	This study
pCDC10	NAT^R^, Amp^R^, *CHO1^R133A^*	This study
pCDC12	NAT^R^, Amp^R^, *CHO1^G142A^*	This study
pCDC11	NAT^R^, Amp^R^, *CHO1^D146A^*	This study
pCDC9	NAT^R^, Amp^R^, *CHO1^D150A^*	This study
pYZ2	NAT^R^, Amp^R^, *CHO1^V180A^*	This study
pYZ3	NAT^R^, Amp^R^, *CHO1^L181A^*	This study
pYZ4	NAT^R^, Amp^R^, *CHO1^C182A^*	This study
pYZ5	NAT^R^, Amp^R^, *CHO1^G183A^*	This study
pYZ6	NAT^R^, Amp^R^, *CHO1^L184A^*	This study
pCDC23	NAT^R^, Amp^R^, *CHO1^R186A^*	This study
pCDC24	NAT^R^, Amp^R^, *CHO1^L187A^*	This study
pCDC22	NAT^R^, Amp^R^, *CHO1^R189A^*	This study
pCDC25	NAT^R^, Amp^R^, *CHO1^F190A^*	This study
pYZ32	NAT^R^, Amp^R^, *CHO1^R133E^*	This study
pYZ50	NAT^R^, Amp^R^, *CHO1^G129P^*	This study

*all CHO1 constructs are tagged on the 3’-terminus with the HAx3 epitope tag sequence.

Site-directed mutagenesis was performed using a primer-based method. Each residue was mutated to alanine in the *CHO1-HAx3* allele carried on the pCDC31 plasmid. Forward and reverse primers approximately 35-40 bp in length were made for each mutation where the codon of interest was modified as conservatively as possible to produce alanine (**Table 4**). Sanger sequencing was used to confirm the alanine substitution on the plasmids generated. The plasmids were then linearized with *MscI* (within the *ENO1* promoter (*P_ENO1_*) sequence) prior to transformation, and integrated into the *P_ENO1_* region in the chromosomal DNA of the *cho1*∆∆ mutant by electroporation. Transformant colonies were selected on YPD plates containing 100 μg/ml nourseothricin. A total of six candidates were chosen for each mutation, and subjected to colony PCR for correct integration. In addition, products from colony PCR for all site-directed mutant candidates were sequenced again to ensure that no spurious mutations had arisen during the transformation.

**Table 4:** Primers used in this study.

Oligonucleotide	Sequence*	Function/Mutation
CCO25	CAGTAAGTTCTTTTAGACTC	Sequencing primer
YZO1	TCAACCACCTTACTCCCTTTATTG	Sequencing primer
CCO160	**aaaaGCGGCCGCATGACAGACTCATCAGCTAC**	Amplifying *CHO1* gene
CCO55	**aaaaGACGTC** ATAGGGATAGCCGGCATAGTCAGGAACATCGTATGGGTAAACGGCCGC ***TGGTTTAGGAATTTTTAAAGAT***	Amplifying *CHO1* gene
CCO56	**aaaaGACGTC** CCGGACTATGCAGGATCCTATCCATATGACGTTCCAGATTACGCTCCGGCCGCC ***TAGAGATGATTCTAAAATAGAAT***	AmplifyingHA tag
CCO163	**aaaaGAGCTCCAGAACCAGAATTATTGTTTC**	AmplifyingHA tag
CCO58	GGGGTTATTTTTCGATTTTTTTGCTGGTAGAGTTGCAAG	D128A
CCO59	CTTGCAACTCTACCAGCAAAAAAATCGAAAAATAACCCC	D128A
CCO60	ATTTTTCGATTTTTTTGATGCTAGAGTTGCAAGATTAAG	G129A
CCO61	CTTAATCTTGCAACTCTAGCATCAAAAAAATCGAAAAAT	G129A
CCO62	TTTTTGATGGTAGAGTTGCAGCTTTAAGAAATAAATCATC	R133A
CCO63	GATGATTTATTTCTTAAAGCTGCAACTCTACCATCAAAAA	R133A
CCO64	ATAAATCATCATTAATGGCTCAAGAGTTAGATTCATTAG	G142A
CCO65	CTAATGAATCTAACTCTTGAGCCATTAATGATGATTTAT	G142A
CCO66	TAATGGGACAAGAGTTAGCTTCATTAGCTGATTTGGTATC	D146A
CCO67	GATACCAAATCAGCTAATGAAGCTAACTCTTGTCCCATTA	D146A
CCO68	GTTAGATTCATTAGCTGCTTTGGTATCATTTGGGGTATC	D150A
CCO69	GATACCCCAAATGATACCAAAGCAGCTAATGAATCTAAC	D150A
CCO82	GTTGGGGTTATTTTTCGCTTTTTTTGATGGTAGAGTTG	D125A
CCO83	CAACTCTACCATCAAAAAAAGCGAAAAATAACCCCAAC	D125A
CCO164	TTTTTGGCCTTTTGGGCTTTATGTGGATTAACAAG	V180A
CCO165	CTTGTTAATCCACATAAAGCCCAAAAGGCCAAAAA	V180A
CCO166	TTGGCCTTTTGGGTTGCATGTGGATTAACAAGATT	L181A
CCO167	AATCTTGTTAATCCACATGCAACCCAAAAGGCCAA	L181A
CCO168	GCCTTTTGGGTTTTAGCTGGATTAACAAGATTGGC	C182A
CCO169	GCCAATCTTGTTAATCCAGCTAAAACCCAAAAGGC	C182A
CCO170	CTTTTGGGTTTTATGTGCATTAACAAGATTGGCTA	G183A
CCO171	TAGCCAATCTTGTTAATGCACATAAAACCCAAAAG	G183A
CCO172	TTGGGTTTTATGTGGAGCAACAAGATTGGCTAGAT	L184A
CCO173	ATCTAGCCAATCTTGTTGCTCCACATAAAACCCAA	L184A
CCO88	GGTTTTATGTGGATTAACAGCTTTGGCTAGATTTAATATC	R186A
CCO89	GATATTAAATCTAGCCAAAGCTGTTAATCCACATAAAACC	R186A
CCO90	GATTAACAAGATTGGCTGCTTTTAATATCTCCGTC	R189A
CCO91	GACGGAGATATTAAAAGCAGCCAATCTTGTTAATC	R189A
CCO94	GGTTTTATGTGGATTAACAAGAGCTGCTAGATTTAATATC	L187A
CCO95	GATATTAAATCTAGCAGCTCTTGTTAATCCACATAAAACC	L187A
CCO96	CAAGATTGGCTAGAGCTAATATCTCCGTCAATAAC	F190A
CCO97	GTTATTGACGGAGATATTAGCTCTAGCCAATCTTG	F190A
YZO28	ATTTTTCGATTTTTTTGATCCAAGAGTTGCAAGATTAAG	G129P
YZO29	CTTAATCTTGCAACTCTTGGATCAAAAAAATCGAAAAAT	G129P
YZO30	TTTTTGATGGTAGAGTTGCAGAATTAAGAAATAAATCATC	R133E
YZO31	GATGATTTATTTCTTAATTCTGCAACTCTACCATCAAAAA	R133E
CCO161	CTTCACTCGATAAGGTGC	Colony PCR
CCO162	AAAAGAGCTCCTAGGCGGCCGGAGCGTAATC	Colony PCR

*Sequences that are bolded on primers CCO160, CCO55, CCO56, and CCO163 hybridize to the CHO1 gene. Sequences that are bolded and underlined are restriction enzyme sites, and run of lower cases aaaa are added to the primer to facilitate cutting.

### Spot Dilution Assay and Growth Curves

To determine ethanolamine auxotrophy, mutant and wildtype (HA1) strains were cultured overnight in liquid YPD medium. The next day, cells were centrifuged and washed three times with water to remove residual nutrients. For spot dilution assays, cells were diluted to OD_600_ = 0.1 using water before three consecutive five-fold dilutions. Then, 10 μl of each dilution was plated onto both minimal medium and minimal medium supplemented with 1 mM ethanolamine. Photos were taken after 24 h incubation at 30°C. For the growth curve, washed cells were diluted to OD_600_ = 0.1 in both liquid minimal medium and minimal medium supplemented with 1 mM ethanolamine. OD_600_ was measured at 2, 4, 6, 8, 10, 12, 24 and 48 hour time points for the growth curve. A total of six biological replicates were measured for OD_600_ for each time point. Doubling time was calculated from the exponential (Malthusian) growth (2-8 h) *via* Graphpad Prism 9.1.

### Western Blots

Protein isolation and western blotting were performed with some modifications to previously published methods (Chen et al., 2019) and according to the manufacturers’ protocols (LI-COR). Cultures were grown overnight in 5 ml YPD, diluted to 0.1 OD_600_/ml in 25 ml of YPD and allowed to grow until reaching early log phase (0.7 – 1 OD_600_/ml). Cultures were centrifuged, washed with water and frozen overnight at -80°C. Pellets were then thawed on ice, resuspended in 1x phosphate buffered saline containing a protease inhibitor cocktail (Roche 4693124001) and lysed with a 200 μl volume of glass beads (Sigma G1145-500G) in a bead-beater at 4°C. Samples were centrifuged at 2,400 x g for 1 minute to clear debris and transferred to a new tube on ice. Again, samples were cleared by centrifuging at 2,400 x g for 8 minutes. This twice-cleared supernatant was moved to fresh tubes on ice and a Bradford assay (Bio-Rad, 5000006) was performed to determine protein concentration.

Proteins were separated by SDS-PAGE (6% stacking/12% separating gel), followed by transfer to PVDF membranes (926-31099 LI-COR). Membranes were dried at room temperature, blocked using TBS: Odyssey Blocking Buffer (1:1) (927-50100 LI-COR) for one hour and incubated overnight at 4˚C in TBS: Odyssey Blocking Buffer (1:1) with primary antibodies (HA-tag monoclonal antibody (26183) and tubulin alpha antibody (MCA78G)) (1:10,000). The following day, after 4 washes in TBST (0.1% Tween 20) at room temperature, membranes were incubated with secondary antibodies (IRDye^®^ 680RD Goat-anti-Rat (926-68076) and IRDye^®^ 800CW Goat-anti-Mouse (926-32210)) (1:10,0000) at room temperature for at least one hour. Membranes were then washed 4x with TBST and imaged using a LI-COR Odyssey scanner. To compare the protein expression of different mutants with respect to HA1, densitometry values from per mutant were quantified against tubulin standards using ImageJ. These tubulin-adjusted densitometry values were further normalized to that of the three HA1 bands together to generate the normalized densitometry values (NDV) of different mutants. The average and standard deviation of the NDVs from two western blots were calculated and shown in **Table S1**.

### *In Vitro* PS Synthase Assay and Calculation of the Adjusted PS Synthase Activity

The cells were grown and broken as previously described in (Cassilly et al., 2017). All strains were grown to OD_600_ between 1.5-2.0 prior to lysis. The cell lysate was cleared by centrifuging at 2000 x g, 4°C for 5 min, then the crude membrane was collected by centrifuging at 27,000 x g, 4°C for 30 min. The pellets were resuspended in 0.1 M Tris-Cl pH 7.5, 5 mM BME, 10% glycerol and protease inhibitors, and the total protein concentration was measured using a Bradford assay.

The PS synthase assay reaction was performed as described in (Cassilly et al., 2017) with the exception that 100 mM Tris-HCl (pH=7.5) and 0.5 mg crude protein were used, and incubation times were set to 30 min. A thirty-minute time point was chosen for single point assays because PS was produced at a constant rate from 0 to 45 min (data not shown), thus a thirty-minute time point allows us to determine the enzymatic activity of Cho1. Briefly, PS synthase activity (nmol/(mg*min)) was measured by monitoring the incorporation of 0.5 mM L-serine spiked with 5% (by volume) L-[^3^H]-serine (30,500 cpm/nmol) in the chloroform phase (for product phosphatidylserine). In an effort to calculate the adjusted PS synthase activity, Cho1 protein expression in the isolated crude membrane was determined using western blotting and was used for normalizing the PS synthase activity. Specifically, the radioactive counts from each mutant, subtracted by the radioactive counts of the *cho1*∆∆ mutant (as the background), were converted to nmol based on the radioactive counts of 1 nmol L-[^3^H]-Serine, and then normalized to 0.5 mg total membrane protein and 30-min reaction time (nmol/(mg*min)). Then this normalized activity of each mutant was further normalized to the relative densitometry values of the Cho1 bands for the final adjusted PS synthase activity. The relative densitometry values were calculated from the densitometry values of each mutant measured from the total membrane prep on the western blot adjusted against that of HA1 *via* ImageJ. Each adjusted PS synthase activity was measured in duplicate with a total of six biological replicates.

### Michaelis-Menten Curves

The Michaelis-Menten curves were generated experimentally based on the *in vitro* PS synthase assay. For the serine Michaelis-Menten curves, the concentration of CDP-DAG was kept at 0.1 mM, and only the initial velocity (calculated based on the rate of linear PS production within 30 min) of Cho1 protein was measured at the serine concentrations of 0.1, 0.5, 1.0, 2.5, 5.0, 7.5, 10 and 15 mM, and then normalized to the relative densitometry values of the Cho1 bands, which are measured from the total membrane prep on the western blot and then adjusted to HA1 *via* ImageJ, for the adjusted specific activity (nmol/(mg*min)). For the CDP-DAG Michaelis-Menten curves, the concentration of serine was kept at 2.5 mM, and the initial velocity of Cho1 protein was measured at the CDP-DAG concentrations of 25, 50, 100, 200, 500, and 1000 μM, and then normalized to the relative densitometry values from the western blotting for the adjusted specific activity. The initial velocity was measured in duplicate with a total of six biological duplicates. The curves were generated and the apparent *K* _m_ and *V*_max_ were estimated using Graphpad Prism 9.1. The statistical comparison of the apparent *K* _m_ and *V*_max_ values were also conducted with Graphpad Prism 9.1 using extra sum-of-squares F test. The ***k*** *_cat_* was calculated using Graphpad Prism 9.1 with the total crude membrane protein serving as the enzyme concentration.

### Homology Modeling

The homology model was produced using Molecular Operating Environment (MOE) software. A Protein Data Bank (PDB) search was performed using the *C. albicans* Cho1 protein sequence and was found to have at least 25% sequence similarity to the four published crystal structures in (Nogly et al., 2014; Sciara et al., 2014; Clarke et al., 2015; Grāve et al., 2019). Several homology models were produced using each of the four crystal structures as templates. The final homology model of Cho1 presented in this manuscript was produced by using the template of the phosphatidylinositolphosphate (PIP) synthase from *Renibacterium salmoninarum* (PDB: 5D92), a CDP-alcohol phosphotransferase, which had approximately 35.3% sequence similarity and 23.1% sequence identity with Cho1 (using the EMBOSS Stretcher alignment tool).

### Statistical Analysis

Statistical analysis for *in vitro* PS synthase assays was performed on Graphpad Prism 9.1 using Brown-Forsythe and Welch ANOVA tests (Assume unequal SDs), and the *post hoc* analysis compares each group to control HA1. Doubling time and the corresponding 95% asymmetrical (profile-likelihood) confidence intervals were calculated by Graphpad Prism 9.1. The best-fit values of the *K* _m_ and *V*_max_ were calculated and compared using Graphpad Prism 9.1.

## Results

The CDP-alcohol phosphatidyltransferase (CAPT) binding motif (D_1_xxD_2_G_1_xxAR … G_2_xxxD_3_xxxD_4_) is highly conserved in enzymes binding CDP-alcohols, even across domains (**Table 1**), indicating the importance of this motif in the function of this type of enzyme. One exception lies in some Gram negative bacteria, such as *E. coli*, where this motif is not present within certain enzymes binding CDP-DAG (e.g., *E. coli* phosphatidylserine synthase, PssA), indicating divergence (Matsumoto, 1997).

Detailed site-directed mutagenesis of the CAPT motif in the *CPT1* cholinephosphotransferase in *S. cerevisiae* (**Table 1**) showed that mutation of Gly114 (G_1_), Gly127 (G_2_), Asp131 (D_3_), or Asp135 (D_4_) caused loss of activity while mutations of Ala117 or Arg118 showed a decrease in activity, and Asp113 (D_2_) showed wildtype activity (Williams and McMaster, 1998). Using *S. cerevisiae* Cpt1 as a guide, we tested whether mutations in amino acids conserved in the CAPT motif of multiple enzymes shown in **Table 1** were important for catalysis in the *C. albicans* PS synthase by mutating them to alanine using site-directed mutagenesis.

**Figure 1:**
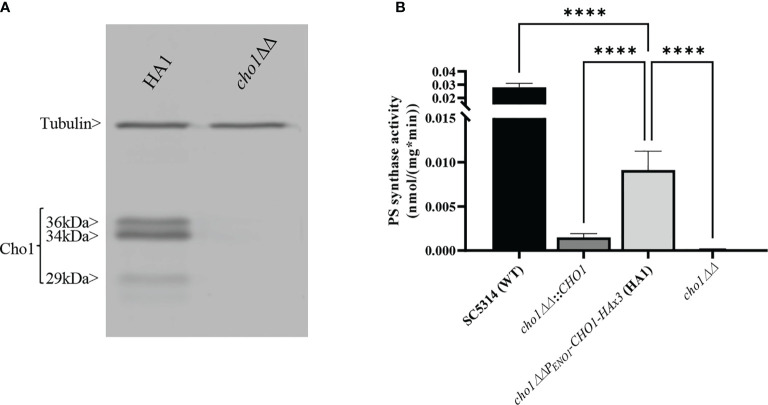
Expression and activity of Cho1-HAx3 under the *ENO1* promoter. **(A)** Proteins were extracted from the *cho1*ΔΔ *P_ENO1_-CHO1-HAx3* (HA1) and *cho1*∆∆ negative control strains, separated on SDS-PAGE and blotted with anti-HA and anti-tubulin (loading control) antibodies. Three bands (36 kDa, 34 kDa and 29 kDa) are present in the HA1 strain, in addition to the tubulin loading control. **(B)** Total membranes were collected from wildtype strain SC5314 (WT), the *CHO1* re-integrated strain where *CHO1* is expressed from its native promoter (*cho1*∆∆*::CHO1*), the HA1 strain, and the *cho1*∆∆ negative control strain. PS synthase activity (nmol/(mg*min)) was measured from 0.5 mg crude membrane protein for 30 min. Statistics were conducted using one-way ANOVA (****p < 0.0001). For each strain, PS synthase activity was measured in duplicate with a total of six biological replicates.

We generated a C-terminally HAx3-tagged version of *C. albicans CHO1* that is transcribed downstream of a constitutive yeast *ENO1* promoter on a SAT1 marked plasmid (pCDC31), and integrated it into the *cho1*∆∆ mutant genome at the *ENO1* locus. Transformants were then screened for expression of Cho1-HAx3 by western blotting, and a successful transformant hereafter known as HA1 (**Figure 1A**) was chosen as the reference strain for this study. It should be noted that three bands (36, 34, and 29 kDa) were observed that were recognized by the anti-HA antibody, but none of these were present in the *cho1*∆∆ control (**Figure 1A**). We suspect that the band at 34 kDa is the full-length protein, which correlates with the predicted molecular weight of 34.8 kDa for Cho1-HAx3 protein. The 36 kDa and 29 kDa bands were shown to contain a phosphorylated protein population as Lambda phosphatase (NEB) decreased the abundance of both bands (**Figure S1**). In addition, the 29 kDa band is likely to be the proteolytic product, as proteolytic maturation of enzymes is a well-documented phenomenon and previous studies reported similar findings with the PS synthase in *S. cerevisiae* (Kinney and Carman, 1988; Kohlwein et al., 1988).These previous studies found that the 30 kDa band was likely proteolytically degraded to produce the 23 kDa band, but both forms of the protein were active (Kinney and Carman, 1988; Kohlwein et al., 1988).

To determine the activity of HAx3-tagged Cho1 under the *ENO1* promoter, PS synthase enzyme activity was directly measured. Membranes were isolated from SC5314 (WT strain), the *CHO1* re-integrated strain (*cho1*∆∆*::CHO1*), the HA1 strain, and the *cho1*∆∆ null mutant. Among these strains, HA1 has restored PS synthase activity compared to the *cho1*ΔΔ strain, and showed ~6 times higher activity than the strain bearing native *CHO1* expressed from its own promoter (*cho1*∆∆*::CHO1*) (**Figure 1B**), indicating that the constitutive *P_ENO1_* promoter significantly increases the expression of Cho1 protein. However, it is noticeable that HA1 strain still has significantly decreased PS synthase activity compared to the WT strain, and the underlying reason is unknown.

### Confirmation of the CAPT Motif’s Importance in Enzyme Activity

**Figure 2:**
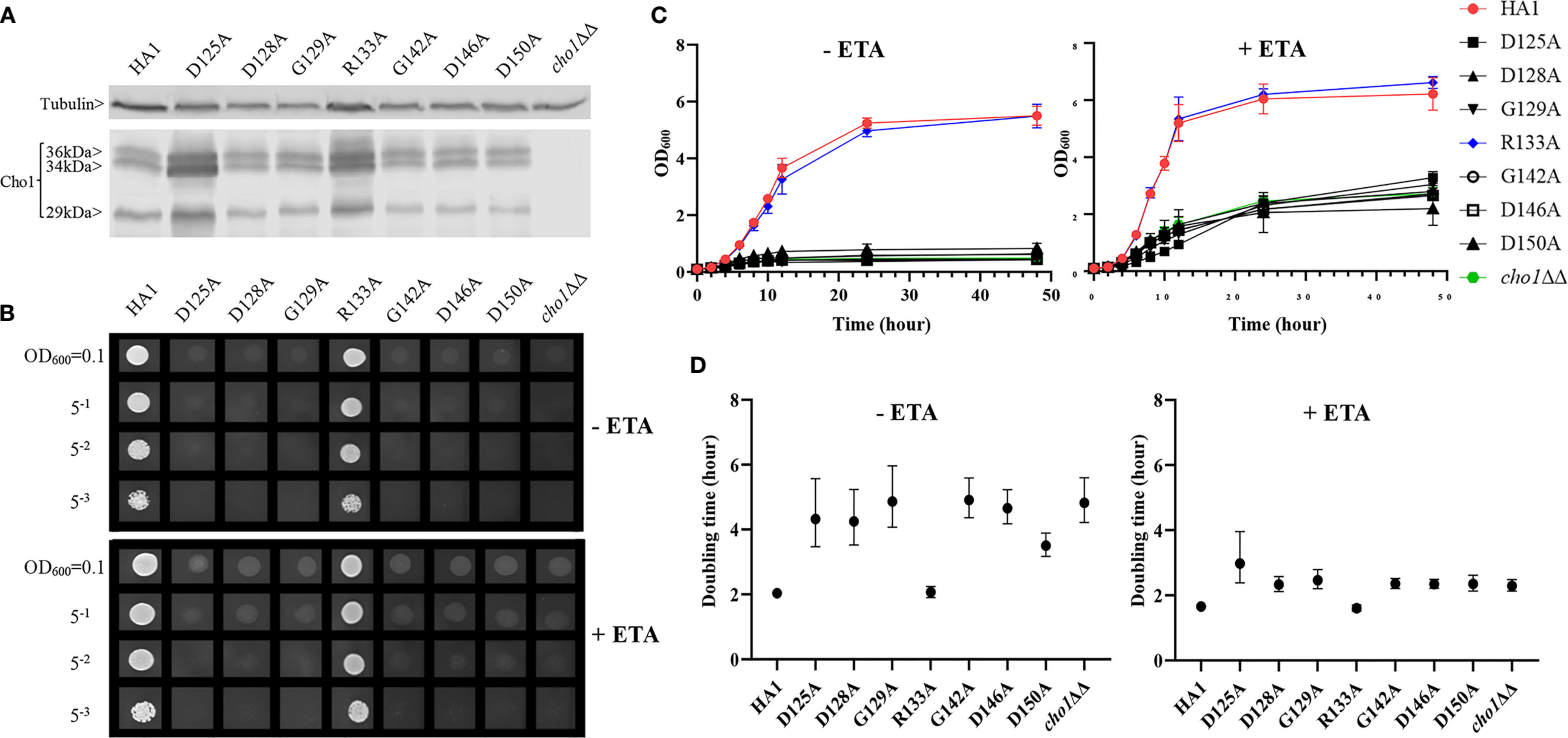
Most conserved CAPT mutants displayed defects in ethanolamine-dependent growth. **(A)** Cho1 protein expression was measured from the cell lysates of *cho1*ΔΔ, HA1, and the CAPT motif mutant strains *via* western blotting. The impact of CAPT mutations on ethanolamine-dependent growth was measured by **(B)** spot dilution assays, **(C)** growth curves, and **(D)** doubling times from corresponding growth curves. Error bars in **(D)** represent the 95% asymmetrical (profile-likelihood) confidence intervals of each doubling time from a total of six replicates. (+ETA, minimal media+1 mM ethanolamine; -ETA, minimal media).

We performed alanine substitution mutagenesis on the conserved non-alanine residues in the CAPT motif to confirm their importance and identify the key residues (**Table 1**, row 1, conserved residues are highlighted in gray). CAPT mutants were made, and a resulting western blot showed that all of the CAPT mutants have comparable Cho1 protein expression to the HA1 wildtype control (**Figure 2A** and **Table S1**). To probe the activities of these mutants, assays were performed to measure PS synthase-dependent phenotypes, including ethanolamine-dependent growth and *in vitro* PS synthase activity.

Mutants that lack the PS synthase have a disruption of the *de novo* pathway for synthesizing phosphatidylethanolamine (PE), an essential phospholipid. In the *de novo* pathway, PE is made by decarboxylating PS; thus, the *cho1*∆∆ mutant relies on exogenous ethanolamine to make PE by a salvage pathway called the Kennedy pathway (Chen et al., 2010). Therefore, these strains show a strong growth perturbation on minimal media with no ethanolamine supplement. For the CAPT mutants, if a mutant grows similarly to the wildtype in the absence of ethanolamine, then its Cho1 function is not disturbed due to the alanine substitution mutations, which indicates that the corresponding original residue is not important in Cho1 function, and *vice versa*. The CAPT mutants, the HA1 strain (positive control), and the *cho1*ΔΔ strain (negative control) were plated on minimal medium without ethanolamine. The R133A mutant grew similarly to HA1, but the other mutants, D125A, D128A, G129A, G142A, D146A and D150A, showed growth perturbations at all cell densities (**Figure 2B**, -ETA). To confirm the growth perturbation is due to the lack of ethanolamine, the CAPT mutants were again grown on minimal medium supplemented with 1 mM ethanolamine, which should give a modest return of growth to the mutants. In the presence of ethanolamine, D125A, D128A, G129A, G142A, D146A and D150A gained obvious growth (**Figure 2B**, +ETA), suggesting the absence of ethanolamine contributes to growth perturbation. However, these mutants still did not grow as well as HA1, which may be explained by inefficient transport of ethanolamine (Davis et al., 2018). To better quantify the impact of Cho1 activity on growth, cells were grown in liquid cultures in the same minimal medium ± 1 mM ethanolamine, and OD_600_ was measured at successive time points (**Figure 2C**). Furthermore, growth curves in the exponential phase (from 2h to 8h) were used to calculate doubling times (**Figure 2D**). Consistent with **Figure 2B**, mutant R133A showed similar growth dynamics to HA1 at different time points and a similar doubling time in the presence and absence of ethanolamine, while the other CAPT mutants had poor growth similar to the *cho1*ΔΔ strain.

**Figure 3:**
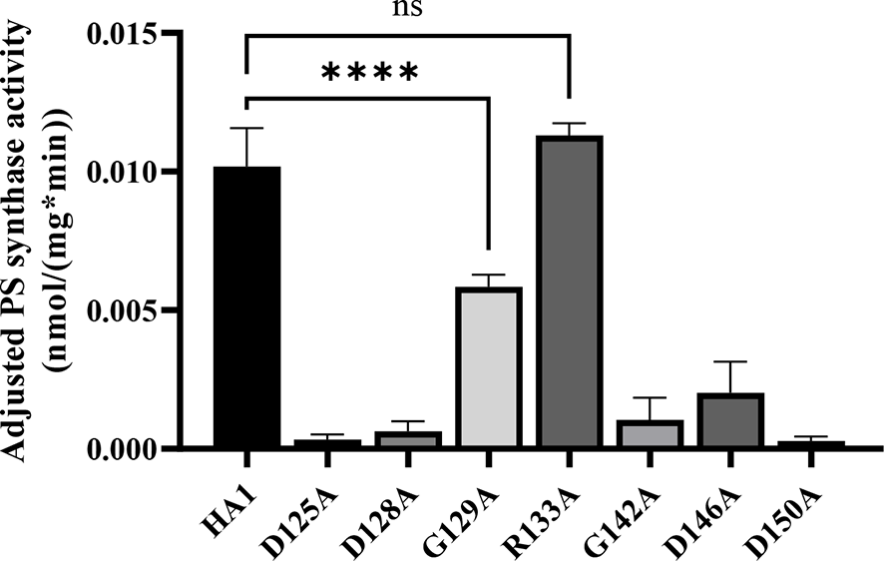
Most of the conserved CAPT motif residues are required for PS synthase activity. Total membranes were collected from *cho1*ΔΔ, HA1, and each CAPT motif mutant and tested in an *in vitro* PS synthase assay. Adjusted PS synthase activity was measured for each total membrane prep. Statistics were conducted using one-way ANOVA, and all mutants were compared to HA1 (****p < 0.0001; ns, not significant). For each strain, the adjusted PS synthase activity was measured in duplicate with a total of six biological replicates.

Alanine substitution of the conserved CAPT residues did not significantly reduce Cho1 protein expression (**Figure 2A** and **Table S1**), but did cause growth perturbations (**Figures 2B–D**) in mutants D125A, D128A, G129A, G142A, D146A and D150A. It was hypothesized that these decreases were due to decreased *in vivo* Cho1 protein function. To test this hypothesis, PS synthase enzyme activities of the CAPT mutants were directly measured. Membranes were isolated from each of the CAPT mutants, along with HA1 and *cho1*∆∆ controls, to assess the enzyme activity of each mutant Cho1 protein using an *in vitro* PS synthase assay. For these reactions, the concentration of crude membrane proteins used from each strain was compared to the estimated level of Cho1 protein expression measured by western blotting to generate a final adjusted PS synthase activity (nmol/(mg*min)). The adjusted PS synthase activities should more accurately reflect the intrinsic enzymatic activity of Cho1 in different strains compared to HA1. In **Figure 3**, the D125A, D128A, G142A, and D150A CAPT mutants showed almost 0% of the activity of the HA1 control while D146A displayed 5-fold lower activity, consistent with the *in vivo* growth assay results (**Figures 2B–D**). R133A mutant retained HA1-level activity which is consistent with its similar growth phenotype compared to HA1 (**Figures 2B–D**). G129A, however, retained almost half the level of adjusted PS synthase activity, which contrasts with its poor growth (**Figures 2B–D**). A possible explanation for this discrepancy is that the level of retained enzymatic activity of G129A is not enough to support the growth under our *in vivo* assay conditions, but it remains detectable in the *in vitro* assay with higher concentrations of protein.

**Figure 4:**
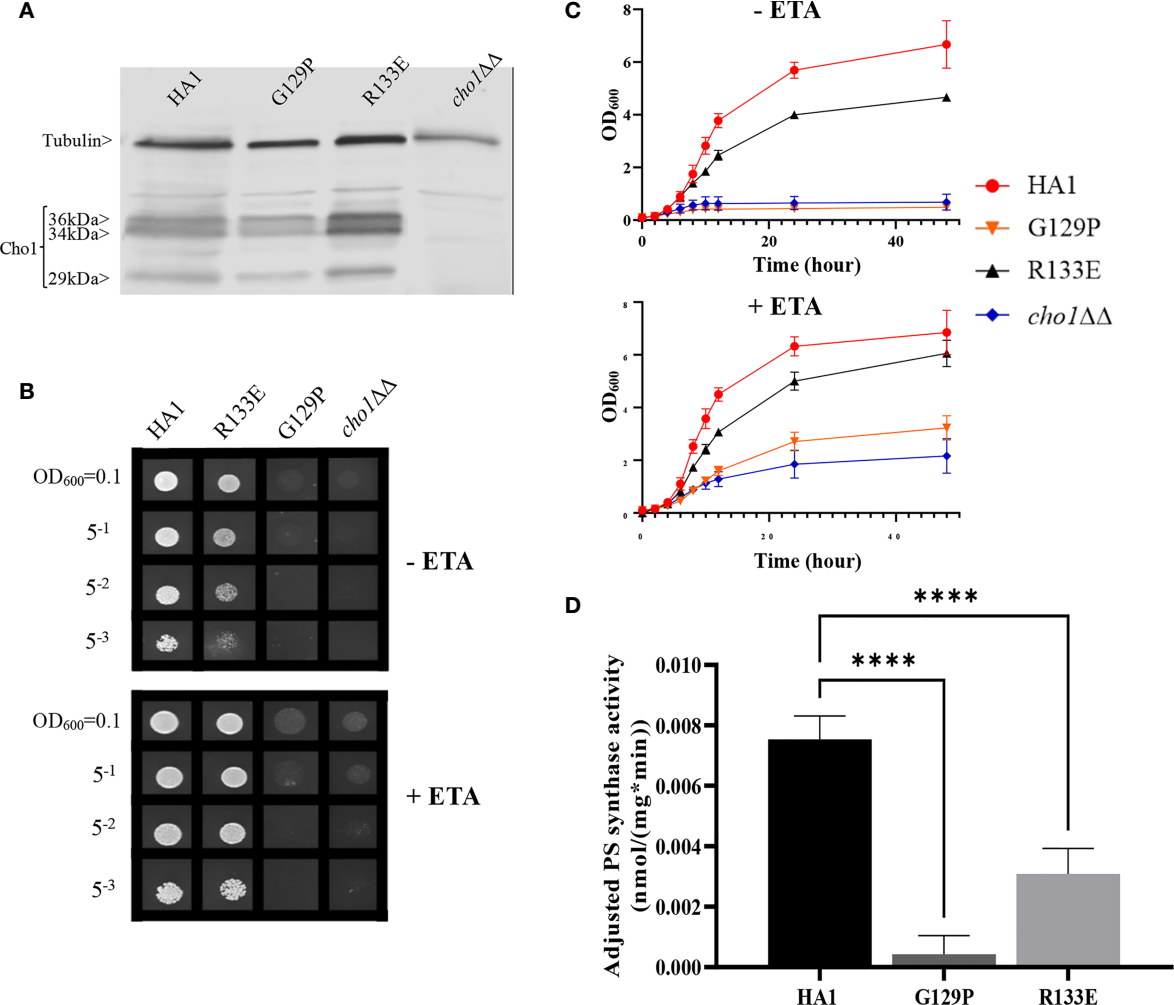
CAPT mutant G129P and R133E displayed decreased Cho1 function. **(A)** Proteins were collected from *cho1*ΔΔ, HA1, G129P and R133E strains, and Cho1 expression was measured using a western blot. Growth was measured by **(B)** spot dilution assays and **(C)** growth curves. **(D)** Adjusted PS synthase activity was measured by the *in vitro* PS synthase assay. Each adjusted PS synthase activity was measured in duplicate with a total of six biological replicates. Statistics was conducted using one-way ANOVA, and all mutants were compared to HA1. ****p < 0.0001 (+ETA, minimal media+1 mM ethanolamine; -ETA, minimal media).

The data from **Figures 2**, **3** demonstrate that the CAPT motif is crucial for PS synthase function. However, there were two main discrepancies in our study compared to previous analyses of the CAPT motif residues: G129 and R133. Our R133A mutant retains HA1-level activity, in contrast with previous studies of similar enzymes bearing CAPT motifs, where the substitution of the conserved arginine residue with either methionine or alanine severely reduced activity (Williams and McMaster, 1998; Nogly et al., 2014). In addition, previous alanine substitution of the first conserved glycine in the CAPT motif in the *S. cerevisiae* cholinephosphotransferase (G129 in *C. albicans* PS synthase numbering) abolished protein expression (Williams and McMaster, 1998), but the *C. albicans* Cho1 G129A protein is expressed and retains partial *in vitro* activity (**Figures 2A** and **3**). To better understand a role for these residues, more drastic substitutions were chosen to replace the two original residues: a negatively charged glutamate substitution was chosen for positively charged arginine (R133), and a rigid proline was chosen for the flexible glycine (G129). We hypothesized that these mutations would result in a marked reduction in Cho1 activity. Hence, mutants R133E and G129P were constructed using the same method above, followed by confirmation of protein expression *via* western blotting (**Figure 4A**). Then, R133E and G129P strains were subjected to subsequent spot dilution assays, growth curve measurements, and *in vitro* PS synthase assays. As predicted, the R133E mutant displayed slower growth compared to HA1 in the absence of ethanolamine (**Figures 4B, C**), in contrast to the R133A mutant (**Figures 2B, C**). The negative effect of R133E is further corroborated by a significant decrease (more than 50%) in adjusted PS synthase activity in the *in vitro* PS synthase assay (**Figure 4D**). The G129P mutation also had a greater impact than the G129A mutation, as the G129P mutant was 17-fold less active than HA1 (**Figure 4D**) compared to G129A, which retained ~50% activity compared to the HA1 strain (**Figure 3**). Thus, these more contrasting substitution mutations suggest that G129 and R133 also play a role in the CAPT motif, but may participate in more flexible interactions with CDP-DAG.

### Analysis of a Predicted Serine Binding Motif

In addition to CDP-DAG, Cho1 also binds serine as a substrate. The serine binding site is more challenging to define compared to the CDP-DAG binding motif since the CDP-DAG motif is common to several other enzymes that bind CDP-alcohols (**Table 1**), whereas the serine binding site is more specific to the Cho1 protein, so limited information is available.

**Figure 5:**
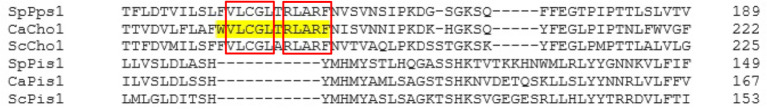
Sequence alignment reveals a possible serine binding motif. Alignment of the PS synthases (Cho1 or Pps1) and PI synthases (Pis1) from *C. albicans* (Ca), *S. cerevisiae* (Sc), and *S. pombe* (Sp) was conducted using Clustal Omega. A highly conserved sequence that is present in Cho1 homologs, but not in the Pis1 homologs, is hypothesized to be part of the serine binding site in PS synthases and is highlighted in yellow in the *C. albicans* sequence. Conserved non-alanine residues in the putative serine binding site are shown in the red boxes.

Here, we took advantage of the fact that the phosphatidylinositol (PI) synthase (Pis1) and PS synthase from yeast are similar enzymes that both bind two substrates: CDP-DAG and a small molecule that serves as the head group of the phospholipid product. Also, PI synthase and PS synthase are reported to use the same sequential reaction mechanism for catalysis (Bae-Lee and Carman, 1984; Fischl et al., 1986). *C. albicans* Pis1 shares 37.2% amino acid similarity (using the EMBOSS Stretcher alignment tool) with Cho1 and only differs enzymatically in binding to inositol instead of serine. We hypothesized that alignment and comparison of sequences between these two genes might reveal a conserved serine binding site in Cho1. Thus, we aligned the Pis1 amino acid sequences from *C. albicans*, *Saccharomyces cerevisiae*, and *Schizosaccharomyces pombe* with the Cho1 amino acid sequence from the same organisms (**Figure 5**). From this alignment, we found a highly conserved sequence in all of the PS synthase amino acid sequences that was absent from the Pis1 sequences (X-V-L-C-G-L-X-R-L-A-R-F). We predicted that this motif might represent part of the serine binding site.

**Figure 6:**
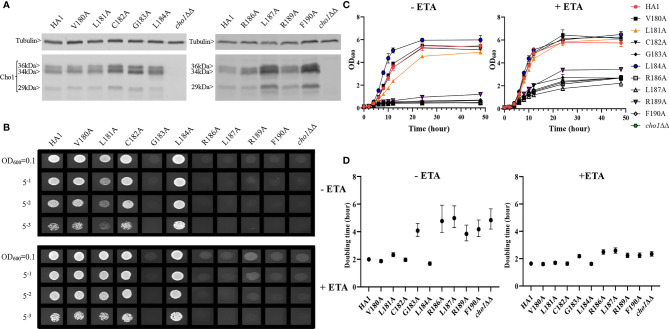
Mutations in the putative serine binding site reduce *in vivo* Cho1 function. **(A)** Cho1 expression from *cho1*ΔΔ, HA1 and each of the putative serine binding site mutants was checked using western blotting. *In vivo* activities were measured in **(B)** spot dilution assays and **(C)** growth curves. **(D)** Doubling times for each strain were calculated from growth curves. Error bars represent the 95% asymmetrical (profile-likelihood) confidence intervals of each doubling time from a total of six replicates. (+ETA, minimal medium+1 mM ethanolamine; -ETA, minimal medium).

Alanine substitution mutagenesis was conducted on the conserved non-alanine residues (**Figure 5**, red boxes) in the putative serine binding site. Putative serine binding site mutants, V180A, L181A, C182A, G183A, L184A, R186A, L187A, R189A and F190A, were constructed and all showed protein expression that is greater than or equal to HA1 (**Figure 6A** and **Table S1**). These mutants were then subjected to *in vivo* spot dilution assays and growth curves. Among all the serine binding site mutants, V180A and C182A had similar growth to HA1 in the absence and presence of ethanolamine, and L181A displayed diminished growth in the absence of ethanolamine, but exhibited restored growth similar to HA1 with the addition of 1 mM ethanolamine (**Figures 6B–D**). The G183A, R186A, L187A, R189A and F190A mutants all displayed diminished growth compared to HA1 in both conditions, but R189A had a greater improvement than the others in the presence of ethanolamine (**Figures 6B–D**). The growth perturbation of these mutants is likely due to significantly decreased *in vivo* Cho1 function. The growth curves and doubling times correlate with the spot dilution assays, where mutants V180A, L181A and C182A showed similar patterns compared to HA1; mutants G183A, R186A, L187A and F190A grew similarly to the *cho1*ΔΔ negative control strain, and mutant R189A had intermediate phenotypes (**Figures 6B–D**).

Surprisingly, L184A demonstrated increased growth in both conditions when compared with HA1 (**Figures 6B**). To rule out the possibility that a spurious mutation might account for the increased growth, several L184A transformation colony candidates were subjected to spot dilution assays and all of them displayed a similar phenotype (**Figure S2**), indicating that the mutation L184A is responsible for the elevated growth. Furthermore, growth curve patterns and shorter doubling times, especially in the absence of ethanolamine (-ETA), are consistent with the spot dilution assay, further supporting the conclusion that L184A leads to elevated growth when compared with HA1 (**Figures 6C, D**). Since mutant L184A does not significantly increase Cho1 expression (**Figure 6A** and **Table S1**), the increased growth of L184A suggests that this mutation increases protein function.

**Figure 7:**
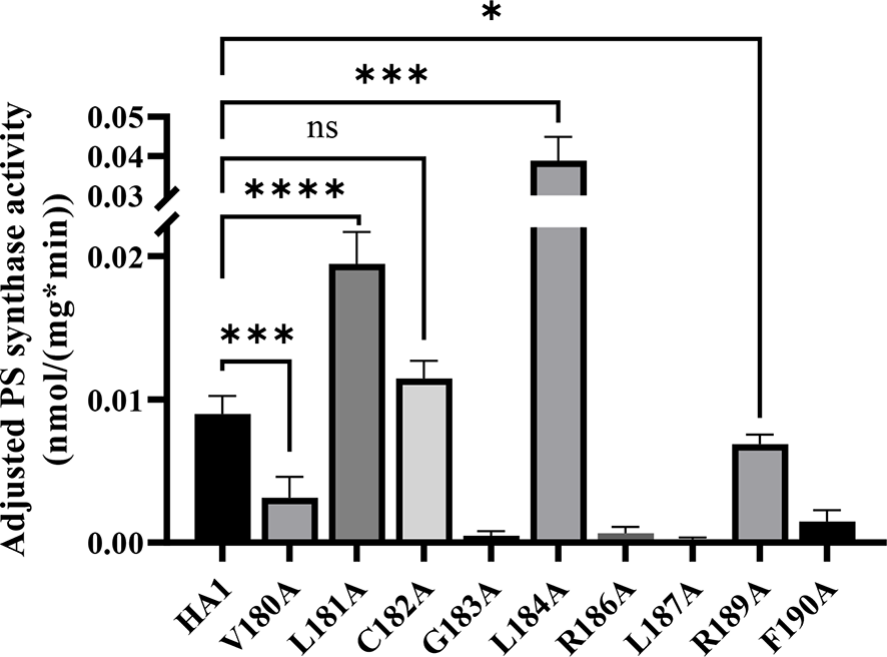
Enzyme activity decreases in some putative serine binding site mutants. Total membranes were collected from *cho1*ΔΔ, HA1 and each of the putative serine binding site mutants and tested in an *in vitro* PS synthase assay. Adjusted PS synthase activity was measured from each total membrane. Statistics were calculated using one-way ANOVA, and all mutants were compared to HA1 (*p < 0.05; ***p < 0.001; ****p < 0.0001; ns, not significant). Each adjusted PS synthase activity was measured in duplicate with a total of six biological replicates.

To further assess these putative serine binding site mutants, we performed the *in vitro* PS synthase assay to measure their adjusted PS synthase activities (**Figure 7**). Consistent with our *in vivo* results, C182A displayed a similar level of activity compared to HA1, while R189A displayed significantly decreased activity. G183A, R186A, L187A and F190A retained almost no activity. In contrast, the hyperactive mutant, L184A, showed significantly enhanced (~5 fold) *in vitro* activity compared to HA1, corroborating the *in vivo* results that mutation L184A increases intrinsic Cho1 activity (**Figures 6** and **Figure S2**).

However, there were two discrepancies between the *in vivo* and *in vitro* results. V180A grew similarly to HA1 in spot assays and growth curves, but yielded significantly lower adjusted PS synthase activity. This discrepancy is likely due to a decreased stability of the V180A mutant protein so that it cannot function properly under our *in vitro* assay conditions. The other inconsistency is L181A, which displayed higher *in vitro* adjusted PS synthase activity but grew similarly to HA1. This can be explained by a more sensitive nature of our *in vitro* PS synthase assay, which is able to detect elevated enzyme activity that is not high enough to support faster growth. Further studies will be needed to conclude the exact nature of these differences. In sum, the putative serine binding site residues, especially the more C-terminal ones, play a role in Cho1 function.

### Enzyme Kinetics Reveal Residue R189 Is Involved in Serine Binding, and L184A Mutation Has Increased *V*_max_

**Figure 8:**
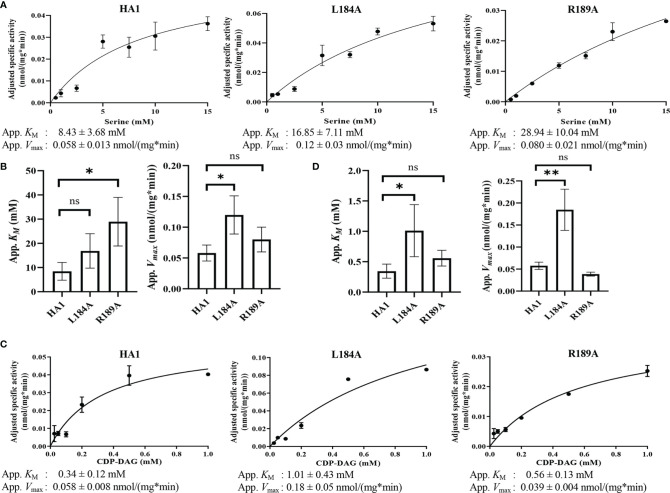
Michaelis-Menten kinetics showed decreased serine binding capacity of R189A. The *in vitro* PS synthase assay was performed with varying concentrations of CDP-DAG and serine, and the Michaelis-Menten kinetics curves of HA1, L184A and R189A were shown in **(A)** for serine and **(C)** for CDP-DAG. The apparent *K*_M_ and *V*_max_ estimated are below the corresponding curves, and are fit into bar graphs **(B)** for serine and **(D)** for CDP-DAG. Statistical comparisons were conducted using extra sum-of-squares F test (*p < 0.1; **p < 0.01; ns, not significant). All adjusted specific activity was measured in duplicate with a total of six biological replicates.

Among the residues in the putative serine binding motif, L184 and R189 are particularly interesting because L184A increases Cho1 activity *in vivo* and *in vitro*, while R189A diminishes activity (**Figures 6**, **7**, respectively). The other mutations: 1) retain original enzyme activity, 2) abolish activity, or 3) display inconsistencies between *in vivo* and *in vitro* activities. Thus, it is of interest to investigate how L184A and R189A alter enzyme kinetics of the Cho1 protein, specifically in regard to the *K* _m_ for serine. For this, Michaelis-Menten curves were produced and kinetic values, apparent *K* _m_ and *V*_max_, of HA1, L184A and R189A were calculated for serine (**Figure 8A**). L-serine, when the concentration of CDP-DAG was held at 0.1 mM, yielded an apparent *K* _m_ of 8.43 ± 3.68 mM and an apparent *V*_max_ of 0.058 ± 0.013 nmol/(mg*min) for HA1, an apparent *K* _m_ of 16.85 ± 7.11 mM and an apparent *V*_max_ of 0.12 ± 0.03 nmol/(mg*min) for L184A, and an apparent *K* _m_ of 28.94 ± 10.04 mM and an apparent *V*_max_ of 0.080 ± 0.021 nmol/(mg*min) for R189A. For comparison, the best-fit values from Graphpad Prism 9.1 of the *K* _m_ and *V*_max_ for HA1, L184A and R189A were used to make bar graphs, with the error bars representing standard errors. Statistics conducted from Graphpad Prism 9.1 (Extra sum-of-squares F test) shows there is a significant increase in the L-serine *K* _m_ for R189A compared to HA1, indicating decreased L-serine binding affinity (**Figure 8B**). This elevated apparent *K* _m_ can also explain the decreased activity of R189A. On the contrary, the increased activity of L184A is not due to a decreased *K* _m_ (thus enhanced L-serine binding), rather it is because of significantly increased apparent *V*_max_ under this condition.

To test whether L184A and R189A affect CDP-DAG binding, Michaelis-Menten kinetics including *K* _m_ and apparent *V*_max_ of HA1, L184A and R189A were calculated for CDP-DAG, where L-serine was held constant at 2.5 mM (**Figure 8C**). Surprisingly, the apparent *K* _m_ for CDP-DAG of L184A was significantly elevated compared to HA1 (**Figure 8D**), indicating a weaker binding. However, the negative effect of L184A for CDP-DAG binding is offset by an elevated *k*_cat_, which leads to a similar *k*_cat_/*K* _m_ compared to HA1 (**Table 5**). In sum, an elevated apparent *V*_max_ of L184A indicates this mutation increases the turnover number, while the increased *K* _m_ of R189A for serine suggests that residue R189 is involved in L-serine binding.

**Table 5:** The *k*_cat_, *K*_M_ and *k*_cat/_ *K*_M_ of HA1, L184A and R189A for both serine and CDP-DAG.

	Serine				CDP-DAG		
	HA1	L184A	R189A		HA1	L184A	R189A
*k_cat_* (h^-1^)	0.12	0.23	0.16	*k_cat_* (h^-1^)	0.12	0.37	0.077
*K _m_ * (mM)	8.43	16.85	28.94	*K _m_ * (mM)	0.34	1.01	0.56
*k_cat_*/*K _m_ * (h^-1^*mM^-1^)	0.014	0.014	0.0055	*k_cat_*/*K _m_ * (h^-1^*mM^-1^)	0.35	0.37	0.138

### Homology Modeling Indicates That the CDP-DAG and L-Serine Binding Motifs Are in Close Proximity

**Figure 9:**
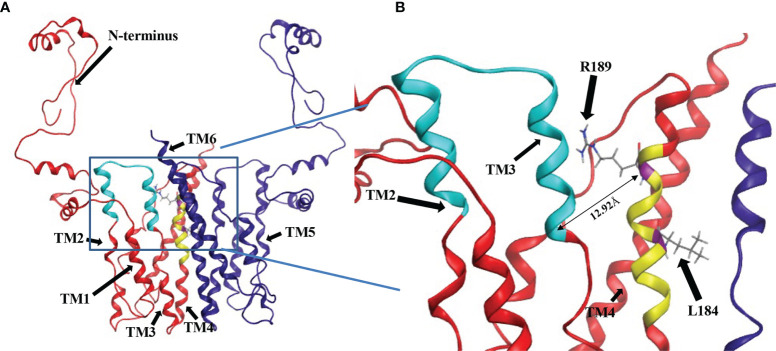
Location of CDP-DAG and putative serine binding residues on a predicted structure of Cho1 based on homology modeling. **(A)** The homology model for the Cho1 protein was built based on the structure of the phosphatidylinositolphosphate (PIP) synthase from *Renibacterium salmoninarum* (PDB: 5D92) using Molecular Operating Environment 2019 software. The two Cho1 monomers are shown in red and dark blue, respectively. The CAPT motif is shown in cyan and the predicted serine binding motif is shown in yellow in the red monomer. TM1, TM2, TM3 and TM4 are indicated in the red monomer, while TM5 and TM6 are indicated in the blue monomer. **(B)** A zoomed-in image of the predicted active site of Cho1 and the locations of residues R189 and L184 on the red monomer is shown. The distance between Cα atoms of residues R189 and D150 is measured at 12.92Å using Molecular Operating Environment (MOE) 2019 software.

Although the mutational evidence suggests that we have identified a motif involved in L-serine binding, its proximity to the CDP-DAG binding CAPT motif was not evident from the primary sequence. Cho1 is a membrane protein and has not yet been crystallized. However, there are several CDP-alcohol phosphotransferase (CDP-AP) proteins containing the CAPT motif from archaea and bacteria that have been crystallized (**Table 1**), and it has been previously suggested that the location of the substrate-binding pocket for the small alcohol molecule is in the proximity of the CAPT motif in the structure (Sciara et al., 2014; Clarke et al., 2015; Grāve et al., 2019; Dufrisne et al., 2020). Four published crystal structures were found *via* Protein Data Bank (PDB) searches using the *C. albicans* PS synthase protein sequence as the query (PDB: 4O6N, 5D92, 6H53 and 4MND) and they all belong to the CDP-AP family (Nogly et al., 2014; Sciara et al., 2014; Clarke et al., 2015; Grāve et al., 2019). An alignment of the four published proteins showed a conserved CAPT binding motif and a similar distribution of secondary structures (data not shown), suggesting high conservation of structure and topology for proteins within this family. Using Molecular Operating Environment software (MOE, Chemical Computing Group, Ltd, Montreal, Canada), the homology model of the *C. albicans* PS synthase was built based on the phosphatidylinositolphosphate (PIP) synthase from *Renibacterium salmoninarum* (PDB: 5D92), which has 35.3% sequence similarity and 23.1% sequence identity with *C. albicans* PS synthase (using the EMBOSS Stretcher alignment tool). The model indicates that *C. albicans* Cho1 forms a homodimer (**Figure 9A**), consistent with the four known CDP-APs (Nogly et al., 2014; Sciara et al., 2014; Clarke et al., 2015; Grāve et al., 2019). Six transmembrane helices (TM1-TM6) are indicated with arrows and an N-terminal cytosolic domain that is connected to TM1. Both the CAPT motif (cyan) and the putative serine binding site (yellow) are highlighted in the red monomer. Specifically, residues L184 and R189 are highlighted in purple and their sidechains are shown (**Figure 9B**). The residues of the putative serine binding site (yellow) in *C. albicans* PS synthase, similarly to the predicted binding pockets of the second small molecules from the four known CDP-APs, are in close enough proximity to the CAPT motif (cyan) to interact during catalysis (**Figure 9A**), further supporting our identified region as the putative serine binding site. Specifically, the side chain of residue R189 projects towards the CAPT motif (**Figure 9B**), and the Cα atomic distance of residue R189 to residue D150 (D_4_), which serves as the catalytic core in some other CDP-APs (Sciara et al., 2014; Grāve et al., 2019), is only 12.92Å, indicating it could coordinate serine and bring it closer to the active site. However, it is likely that other parts of the protein also help to coordinate serine, and this represents only part of the overall serine binding site. Further studies will be required to provide greater insight into all residues involved in the binding and positioning of serine during catalysis.

## Discussion

In this study, we found that most of the conserved amino acids (**Table 1**) in the CAPT motif in *C. albicans* Cho1 are necessary for PS synthesis (**Figures 2**, **3**). However, there were differences from what has been observed in the CAPT motif for some other enzymes. For example, as opposed to the findings in *S. cerevisiae* Cpt1 (**Figure S3**), mutations of G129 and D146 showed a severe to a modest decrease in activity, and R133 showed nearly wildtype level activity (**Figure 3**). In the Cpt1 enzyme from *S. cerevisiae*, replacing G114 (equivalent to G129) and D131 (equivalent to D146) with alanine abolished enzyme activity, while the R118A (equivalent to R133A) mutant displayed decreased activity (Williams and McMaster, 1998). It was suggested that the importance of the first glycine (G114 or G129) in the CAPT motif is due to binding or positioning of the CDP-alcohol (Williams and McMaster, 1998). This statement is further supported by a solved structure of AF2299, a representative CDP-AP from *Archaeoglobus fulgidus* (PDB: 4O6N), where the first glycine in the motif provides flexibility to TM2 for catalysis (Sciara et al., 2014). Here, we made a proline substitution mutation, G129P, which almost abolished activity (**Figure 4**), suggesting that the flexibility provided by this glycine is essential for catalysis of *C. abicans* Cho1. Furthermore, the arginine residue within the CAPT motif was shown to be important in binding and positioning of the CDP-alcohol substrate *via* electrostatic interactions (Williams and McMaster, 1998; Sciara et al., 2014), but our R133A showed unchanged activity compared to HA1 (**Figures 2**, **3**). However, a reversal of the charge by the R133E mutation significantly decreased activity compared to HA1 (**Figure 4**), indicating the arginine residue in the CAPT motif of *C. albicans* Cho1 potentially binds, or at least is in the proximity of, the CDP-DAG molecule, but that this interaction is not absolutely required. We hypothesize that the loss of the R133 residue can be compensated for by the adjacent positively charged R131 or K135 residue in the *C. albicans* Cho1 sequence. The four aspartic acid residues within the CAPT motif point to the same patch of electron density and likely participate in cofactor cation binding and catalysis that is shown in currently published structures (Nogly et al., 2014; Sciara et al., 2014; Clarke et al., 2015; Grāve et al., 2019; Dufrisne et al., 2020). These residues (D125, D128, D146 and D150, *C. abicans* Cho1 numbering) are also important in *C. abicans* Cho1 (**Figures 2**, **3**), indicating a similar function.

In addition, we predicted a serine binding motif, X-V-L-C-G-L-X-R-L-A-R-F, based on sequence alignment from three different yeasts, which is present in all three fungal PS synthase sequences, but absent from all three PI synthase sequences (**Figure 5**). Interestingly, a further alignment of 8 previously reviewed PS synthases with 7 PI synthase sequences from a variety of species including the *C. albicans* Cho1 sequence also generates part of the predicted serine binding motif (**Figure S4**), especially the C-terminal section. Site-directed mutagenesis of the residues within the serine binding motif in the *C. albicans* Cho1 protein showed that three of the C-terminal four residues (R186A, L187A and F190A) produced an almost complete abolition of activity while R189A displayed significantly decreased activity (**Figures 6**, **7**), supporting the hypothesis that these residues play roles in Cho1 catalytic function. Moreover, after this paper was accepted for publication, Centola et al published the structure of a phosphatidylserine synthase from the archaeon *Methanocaldococcus jannaschii*, and showed this motif is involved in coordinating serine (Centola et al., 2021). 

Furthermore, we have found that the decreased activity of the R189A mutant is due to the significantly decreased binding affinity for serine (as reflected by increased apparent *K* _m_ , **Figure 8B**), suggesting the residue R189 is involved in serine binding. This is also supported by the position of residue R189 in the homology model of Cho1, which is in proximity to the CAPT motif (e.g., the distance of Cα from R189 and D150 is 12.92Å), and thus could bind and position serine for catalysis (**Figure 9B**). Further investigation will be required to determine if this interaction is occurring between R189 and serine in the fungal PS synthase. Finally, L184A demonstrated a significantly increased *V*_max_, but also increased *K* _m_ for CDP-DAG (**Figure 8D**). The similar specificity constants (*k*_cat_/*K* _m_ ) of L184A with respect to HA1 suggest that mutation L184A does not make Cho1 use either substrate more efficiently (**Table 5**), but rather the effect of increased *V*_max_ outcompetes that of an increased *K* _m_ , leading to a net outcome of increased activity. This also indicates that the turnover number (*k*_cat_), rather than substrate binding (*K* _m_ ), is the limiting factor for the catalysis of Cho1 protein. Currently, the function of residue L184 is unknown.

In order to determine the relative locations of the putative serine binding site and the CAPT motif, we produced a homology model of Cho1. The homology model of *C. albicans* PS synthase was built based on the phosphatidylinositolphosphate (PIP) synthase from *Renibacterium salmoninarum* (PDB: 5D92) and provided a preliminary view of what Cho1 might look like (**Figure 9**). The model predicts a six-transmembrane domain protein structure where the CAPT and predicted serine binding motifs are within relatively close proximity to one another. Many of the residues within the CAPT motif are charged and have been found to be part of a hydrophilic face of the 2^nd^ and 3^rd^ transmembrane (TM2/3) domains of this protein, correlating well with the current structures (Nogly et al., 2014; Sciara et al., 2014; Clarke et al., 2015; Grāve et al., 2019; Dufrisne et al., 2020). The predicted serine binding motif (yellow) is located on TM4 and is spatially in the interface of cytosolic and membrane-bound portions, possibly allowing serine entry into this site. This correlates well with the previous findings that the second small molecules for CDP-AP proteins (serine, in the example of Cho1) bind in a cavity formed by TM 4, 5 and 6 not deeply in the membrane (Nogly et al., 2014; Clarke et al., 2015; Grāve et al., 2019). This, again, indicates that there are additional residues involved in serine binding.

We have demonstrated here, for the first time, the importance of the CAPT motif within the PS synthase from a medically relevant, pathogenic fungus. Furthermore, we have begun efforts to identify the binding site for the second substrate in this enzyme, serine, an area that is still relatively unstudied. We have produced a homology model of Cho1 that places both of the substrate binding sites in close proximity and lays the foundation for structural studies. These findings contribute not only to the general understanding of phospholipid synthesizing enzymes, but also provide crucial information on candidate locations in this protein where binding of small molecule drug candidates may interfere with substrate binding. However, a limitation of this study is that it did not investigate the impact of mutations on post-translational regulation of Cho1. For example, further experiments will be required to determine whether CAPT and serine binding mutants of Cho1 have different subcellular localizations compared to HA1, as membrane localization affects Cho1 function (Kohlwein et al., 1988). In addition, phosphorylation has been shown to regulate *S. cerevisiae* Cho1 protein by inhibiting enzymatic activity, but retains normal Cho1 protein level during exponential and stationary growth phases (Kinney and Carman, 1988; Choi et al., 2010). In this study, the phosphorylation level variations among different mutants were not taken into account for the *in vitro* PS synthase assay as we do not know what effect phosphorylation of Cho1 has in *C. albicans*. The impact of variation was minimized by growing the different strains to a similar OD_600_ of 1.5-2.0 in the early exponential phase, in which the PS synthesis activity was maximal (Homann et al., 1987).

Cho1 represents a promising drug target, so an additional, valuable data set will come from exploring how the mutants that impact enzyme catalysis affect virulence. This data should lead to a more precise definition of how much enzymatic inhibition is required to cause a loss of virulence and better direct future inhibitor studies.

## Data Availability Statement

The original contributions presented in the study are included in the article/**Supplementary Material**. Further inquiries can be directed to the corresponding author.

## Author Contributions

Conceived and designed the experiments: YZ, CC, and TR. Performed the experiments: YZ and CC. Analyzed the data: YZ and TR. Contributed reagents/materials/analysis tools: TR. Wrote the paper: YZ, CC, and TR. All authors contributed to the article and approved the submitted version.

## Funding

This work was supported by the National Institutes of Health (grant numbers: NIH-1 R01AI153599 (TBR) and NIH 1R21AI130895-01 (TBR)). The funders had no role in study design, data collection and interpretation, or the decision to submit the work for publication.

## Author Disclaimer

The content is solely the responsibility of the authors and does not necessarily represent the official views of the National Institutes of Health.

## Conflict of Interest

The authors declare that the research was conducted in the absence of any commercial or financial relationships that could be construed as a potential conflict of interest.

## Publisher’s Note

All claims expressed in this article are solely those of the authors and do not necessarily represent those of their affiliated organizations, or those of the publisher, the editors and the reviewers. Any product that may be evaluated in this article, or claim that may be made by its manufacturer, is not guaranteed or endorsed by the publisher.
