# Supplementary material for: Mapping the Substrate-Binding Sites in the Phosphatidylserine Synthase in Candida albicans
Source: Front Cell Infect Microbiol. 2021 Dec 22;11:765266. doi: 10.3389/fcimb.2021.765266 (PMC8727905; doi:10.3389/fcimb.2021.765266)
Supplement: Supplementary file 1 [file Presentation_1.pptx]

## Slide 1
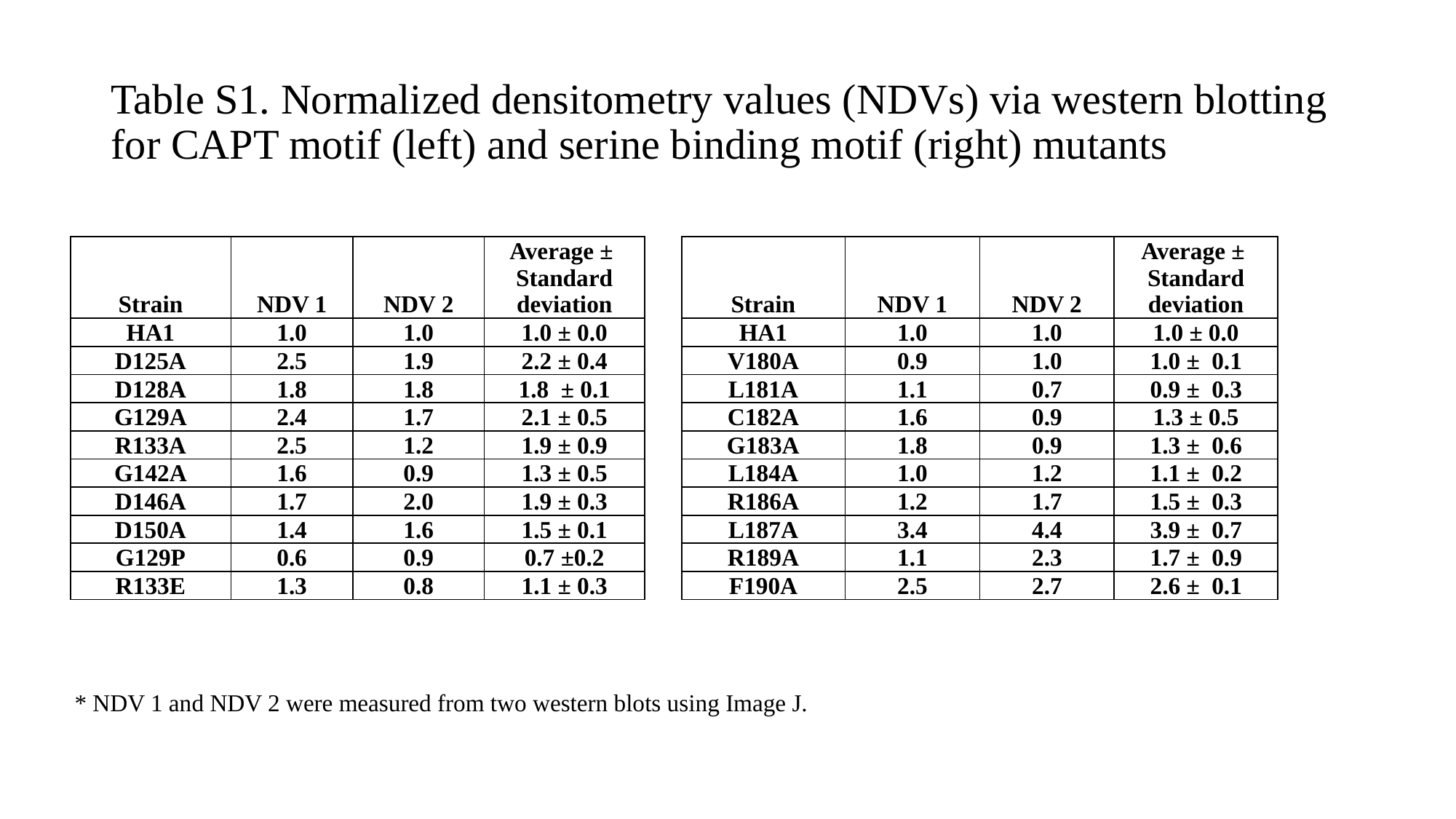

# Table S1. Normalized densitometry values (NDVs) via western blotting for CAPT motif (left) and serine binding motif (right) mutants
| Strain | NDV 1 | NDV 2 | Average ± Standard deviation |
| --- | --- | --- | --- |
| HA1 | 1.0 | 1.0 | 1.0 ± 0.0 |
| D125A | 2.5 | 1.9 | 2.2 ± 0.4 |
| D128A | 1.8 | 1.8 | 1.8 ± 0.1 |
| G129A | 2.4 | 1.7 | 2.1 ± 0.5 |
| R133A | 2.5 | 1.2 | 1.9 ± 0.9 |
| G142A | 1.6 | 0.9 | 1.3 ± 0.5 |
| D146A | 1.7 | 2.0 | 1.9 ± 0.3 |
| D150A | 1.4 | 1.6 | 1.5 ± 0.1 |
| G129P | 0.6 | 0.9 | 0.7 ±0.2 |
| R133E | 1.3 | 0.8 | 1.1 ± 0.3 |
| Strain | NDV 1 | NDV 2 | Average ± Standard deviation |
| --- | --- | --- | --- |
| HA1 | 1.0 | 1.0 | 1.0 ± 0.0 |
| V180A | 0.9 | 1.0 | 1.0 ± 0.1 |
| L181A | 1.1 | 0.7 | 0.9 ± 0.3 |
| C182A | 1.6 | 0.9 | 1.3 ± 0.5 |
| G183A | 1.8 | 0.9 | 1.3 ± 0.6 |
| L184A | 1.0 | 1.2 | 1.1 ± 0.2 |
| R186A | 1.2 | 1.7 | 1.5 ± 0.3 |
| L187A | 3.4 | 4.4 | 3.9 ± 0.7 |
| R189A | 1.1 | 2.3 | 1.7 ± 0.9 |
| F190A | 2.5 | 2.7 | 2.6 ± 0.1 |
* NDV 1 and NDV 2 were measured from two western blots using Image J.

## Slide 2
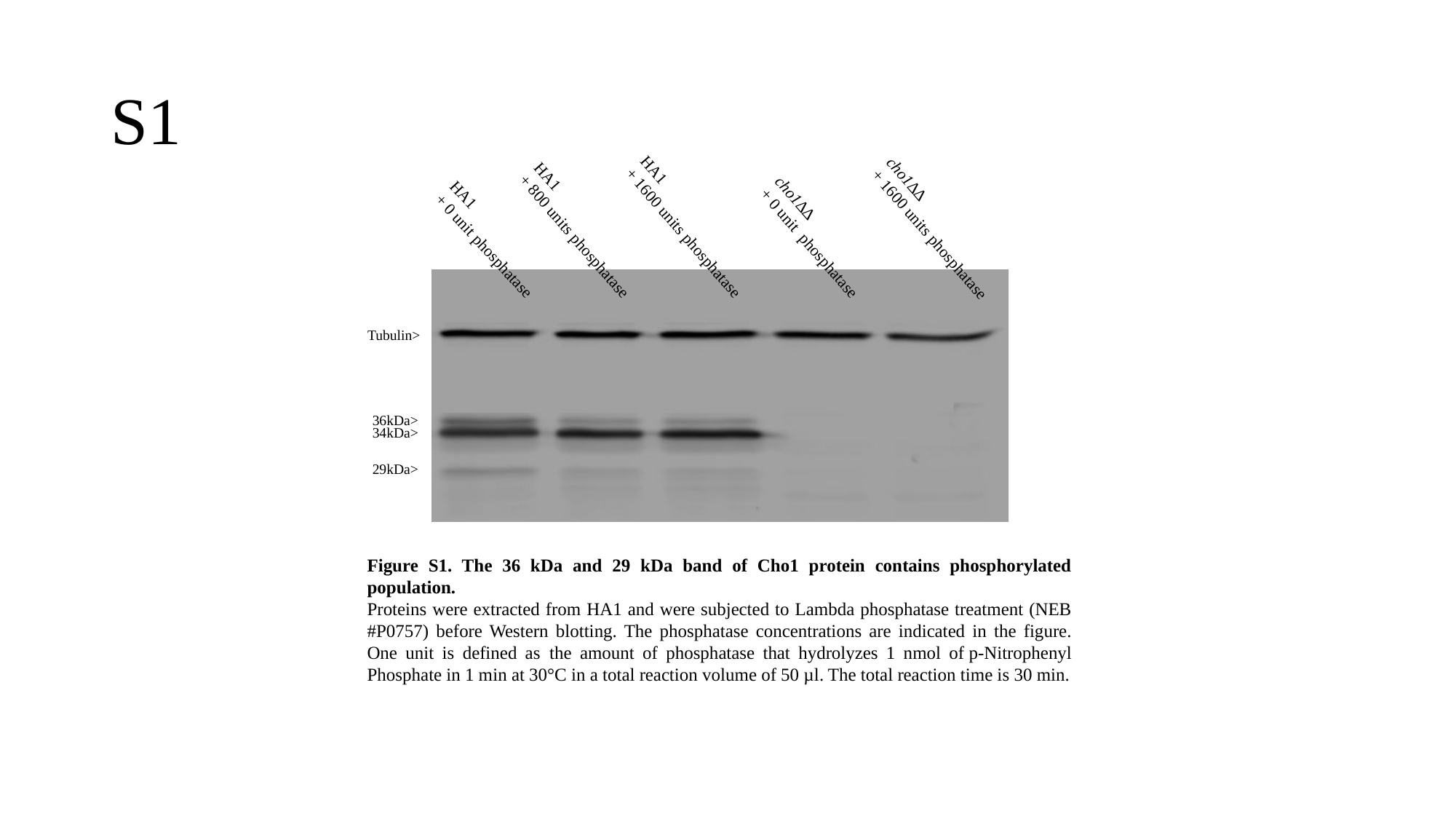

# S1
HA1
+ 1600 units phosphatase
cho1∆∆
+ 1600 units phosphatase
HA1
+ 800 units phosphatase
cho1∆∆
+ 0 unit phosphatase
HA1
+ 0 unit phosphatase
Tubulin>
36kDa>
34kDa>
29kDa>
Figure S1. The 36 kDa and 29 kDa band of Cho1 protein contains phosphorylated population.
Proteins were extracted from HA1 and were subjected to Lambda phosphatase treatment (NEB #P0757) before Western blotting. The phosphatase concentrations are indicated in the figure. One unit is defined as the amount of phosphatase that hydrolyzes 1 nmol of p-Nitrophenyl Phosphate in 1 min at 30°C in a total reaction volume of 50 µl. The total reaction time is 30 min.

## Slide 3
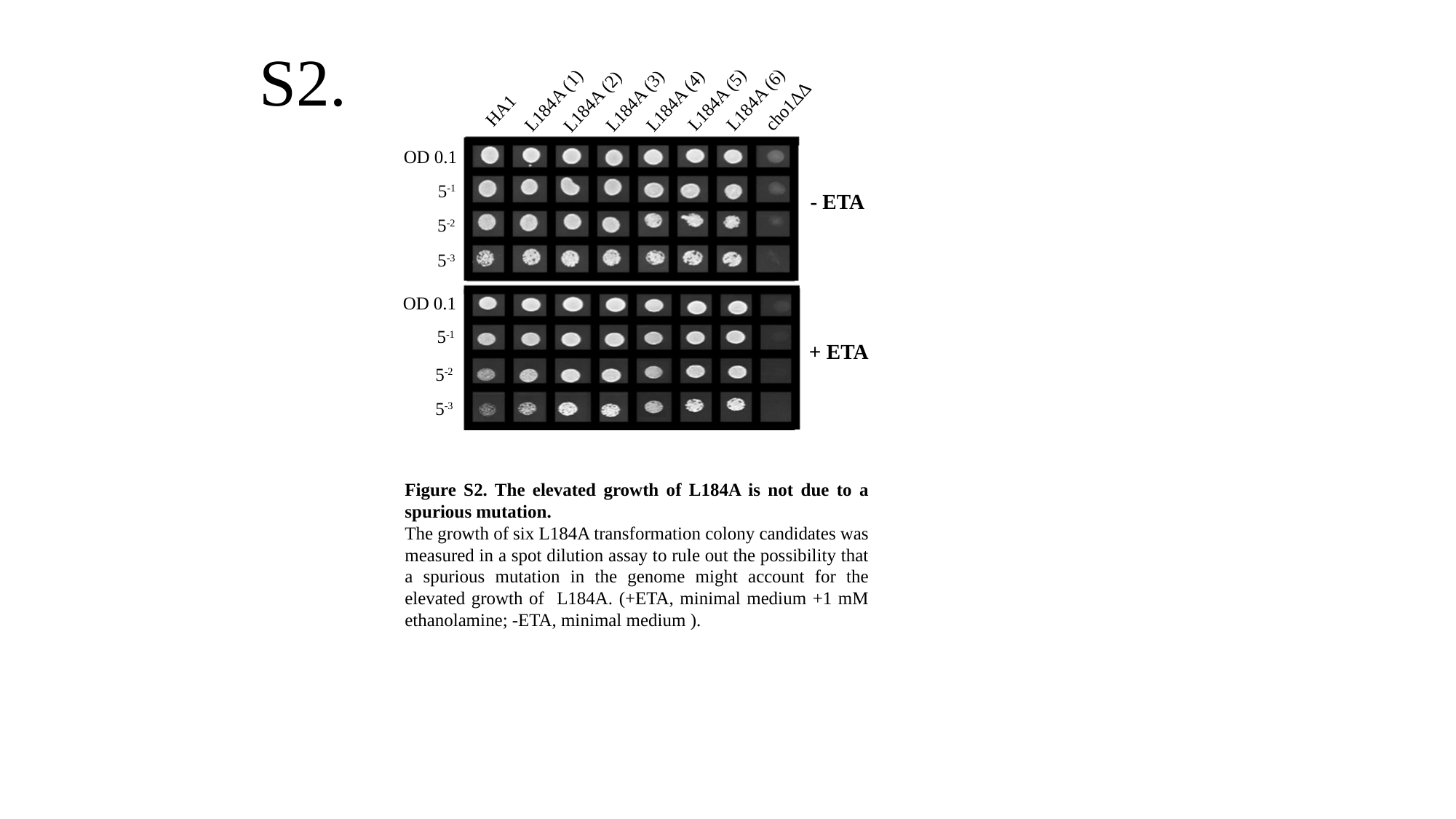

# S2.
L184A (5)
L184A (6)
L184A (1)
L184A (3)
L184A (4)
L184A (2)
cho1ΔΔ
HA1
OD 0.1
5-1
5-2
5-3
OD 0.1
5-1
5-2
5-3
- ETA
+ ETA
Figure S2. The elevated growth of L184A is not due to a spurious mutation.
The growth of six L184A transformation colony candidates was measured in a spot dilution assay to rule out the possibility that a spurious mutation in the genome might account for the elevated growth of L184A. (+ETA, minimal medium +1 mM ethanolamine; -ETA, minimal medium ).

## Slide 4
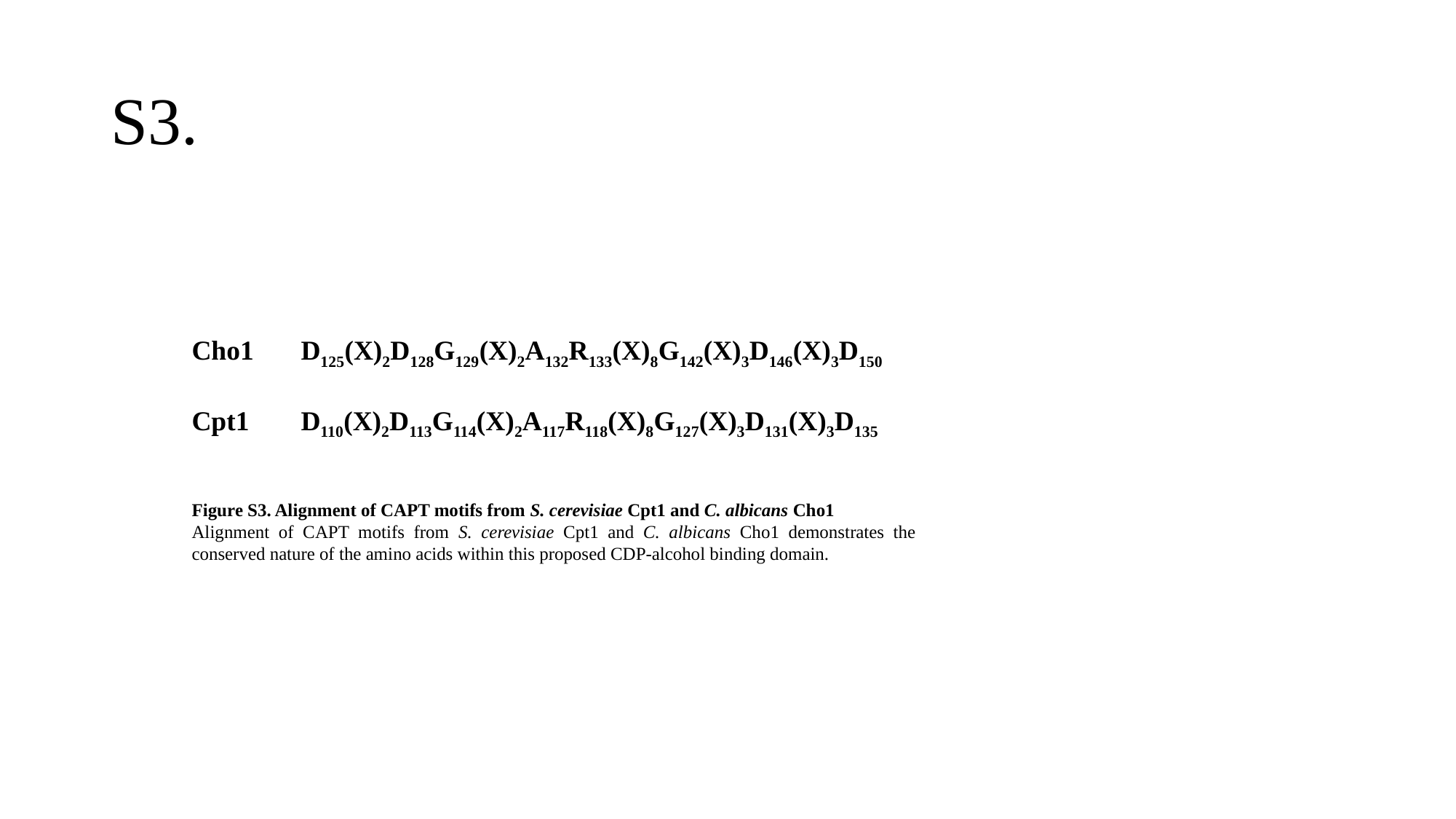

# S3.
Cho1	D125(X)2D128G129(X)2A132R133(X)8G142(X)3D146(X)3D150
Cpt1	D110(X)2D113G114(X)2A117R118(X)8G127(X)3D131(X)3D135
Figure S3. Alignment of CAPT motifs from S. cerevisiae Cpt1 and C. albicans Cho1
Alignment of CAPT motifs from S. cerevisiae Cpt1 and C. albicans Cho1 demonstrates the conserved nature of the amino acids within this proposed CDP-alcohol binding domain.

## Slide 5
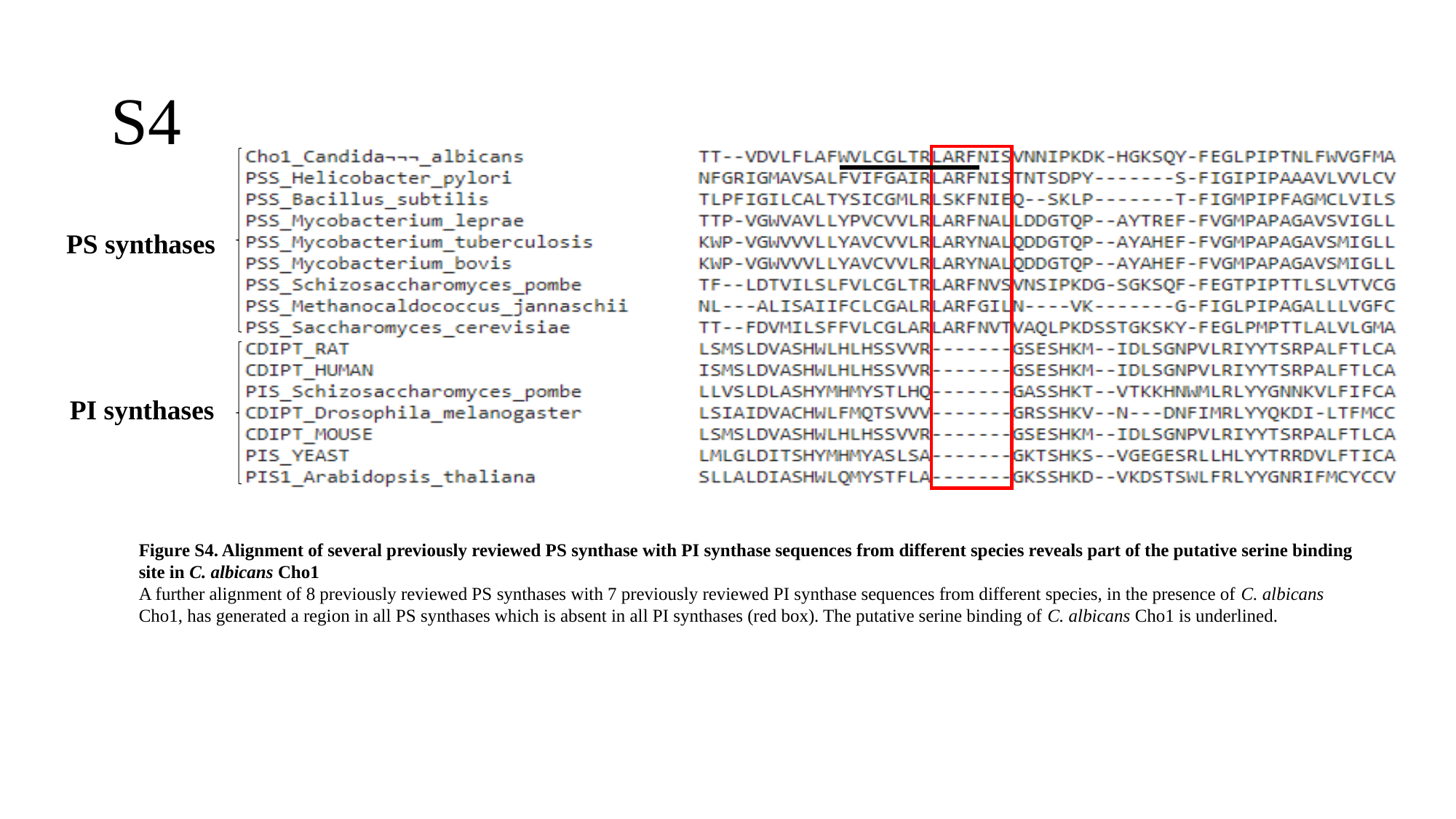

# S4
PS synthases
PI synthases
Figure S4. Alignment of several previously reviewed PS synthase with PI synthase sequences from different species reveals part of the putative serine binding site in C. albicans Cho1
A further alignment of 8 previously reviewed PS synthases with 7 previously reviewed PI synthase sequences from different species, in the presence of C. albicans Cho1, has generated a region in all PS synthases which is absent in all PI synthases (red box). The putative serine binding of C. albicans Cho1 is underlined.
